# Development of novel CDK9 and CYP3A4 inhibitors for cancer therapy through field and computational approaches

**DOI:** 10.3389/fchem.2024.1473398

**Published:** 2024-10-21

**Authors:** Aisha A. Alsfouk, Abdelmoujoud Faris, Ivana Cacciatore, Radwan Alnajjar

**Affiliations:** ^1^ Department of Pharmaceutical Sciences, College of Pharmacy, Princess Nourah bint Abdulrahman University, Riyadh, Saudi Arabia; ^2^ LIMAS, Department of Chemical Sciences, Faculty of Sciences Dhar El Mahraz, Sidi Mohamed Ben Abdellah University, Fez, Morocco; ^3^ Department of Pharmacy, University “G. d’Annunzio” of Chieti-Pescara, Chieti, Italy; ^4^ CADD Unit, Faculty of Pharmacy, Libyan International Medical University, Benghazi, Libya

**Keywords:** drug design, CADD, CDK9, Cancer, 3D-QSAR

## Abstract

Cyclin-dependent kinase 9 (CDK9) and cytochrome P450 3A4 (CYP3A4) have emerged as promising targets in the development of anticancer drugs, presenting a consistent challenge in the quest for potent inhibitors. CDK9 inhibitors can selectively target fast-growing cancer cells by disrupting transcription elongation, which in turn hinders the production of proteins essential for cell cycle progression and survivaŚ. Understanding how CYP3A4 metabolizes specific chemotherapy drugs allows for personalized treatment plans, optimizing drug dosages according to a patient’s metabolic profile. Since many cancer patients undergo combination therapies, and CYP3A4 is vital in drug metabolism, its inhibition or induction by one drug can alter the plasma levels of others, potentially leading to treatment failure or increased toxicity. Therefore, managing CYP3A4 activity is critical for effective cancer treatment. Employing a range of computational methodologies, this study systematically investigated the binding mechanisms of pyrimidine derivatives against CDK9 and CYP3A4. The field-based model demonstrated high *R* ^2^ values (0.99), with Q^2^ (0.66), demonstrating its ability to predict *in silico* inhibitory activity against the target of this study. The screening process followed in this work led to the discovery of powerful new inhibitor compounds. Of the 15 new compounds designed, three have a high affinity with the target (ranging from −8 to −9 kcal/mol kcal/mol) and were singled out through docking filtration for more detailed investigation. As well as, a reference compound with a substantial pIC_50_ value of 8.4, serving as the foundation for the development of the new compounds, was included for comparative analysis. To elucidate the essential features of CDK9 and CYP3A4 inhibitor design, a comparative analysis was conducted between 3D-QSAR-generated contours and molecular docking conformations of ligands. Molecular dynamics simulations were carried out for a duration of 100 ns on selected docked complexes, specifically those involving novel compounds with CDK9 and CYP3A4 enzymes. Additionally, the binding free energy for these complexes was assessed using the MM/PBSA method, which evaluates the free energy landscape of protein-ligand interactions. The results of MM/PBSA highlighted the strength of the new compounds in enhancing interactions with the target protein, which favors the results of molecular docking and MD simulation. These insights contribute to a deeper understanding of the mechanisms underlying CDK9 and CYP3A4 inhibition, offering potential avenues for the development of innovative and effective CDK9 inhibitors.

## Introduction

Epigenetic mutations are important for the onset and spread of cancer (Baylin and Jones, 2016; Jones et al., 2016; Alsfouk et al., 2022). The aging process and the accumulation of genetic and epigenetic modifications can lead to changes in gene expression, including the silence of tumor suppressor genes (Issa, 2014; Alsfouk et al., 2023). When methyl-binding proteins are attracted to the promoters of tumor suppressor genes, DNA methylation frequently results in the silence of those genes (Raynal et al., 2012; Duan et al., 2022). These proteins then attract repressor complexes, which lead to the creation of heterochromatin and the preservation of gene inactivation (Nan et al., 1998).

Cyclin-dependent kinases (CDKs), including CDK7 and CDK9, play a pivotal role in regulating transcription (Malumbres, 2014). CDK9, as part of the P-TEFb complex, functions as a transcriptional kinase by phosphorylating negative elongation factors. This phosphorylation facilitates the release of promoter-proximal stalled RNA polymerase II, thereby enabling transcriptional elongation. (Adelman and Lis, 2012; Budhiraja et al., 2013). Recruitment of RNA processing components is thus made possible by CDK9’s phosphorylation of RNA polymerase II’s C-terminal domain (Church et al., 2017; Eyvazi et al., 2019). Therefore, numerous genes involved in essential activities, including stress response, survival, and proliferation, are encouraged to elongate through the action of CDK9 (Garriga et al., 2003; Bacon and D’Orso, 2019). Its overexpression or hyperactivation can result in unchecked cell proliferation, a key feature of cancer. Thus, targeting CDK9 presents a potential therapeutic strategy to inhibit tumor growth by interfering with the specific DNA programs that drive oncogenesis. CYP3A4 (Cytochrome P450 Family 3 Subfamily A Member 4) is a key enzyme involved in the metabolism of various drugs, including chemotherapeutic agents (Alsfouk et al., 2023). In cancer, CYP3A4 can affect the efficacy of chemotherapy by metabolizing anticancer drugs, which may lead to drug resistance. Additionally, alterations in CYP3A4 expression in cancerous tissues can influence the bioavailability and therapeutic effectiveness of treatments (Marra and Curigliano, 2019; Roncato et al., 2020). Targeting CYP3A4 has the potential to enhance the sensitivity of tumors to chemotherapy and counteract resistance mechanisms. Although CDK9 and CYP3A4 perform distinct biological functions, targeting both could yield synergistic effects in cancer treatment. Inhibiting CDK9 disrupts transcriptional processes crucial for cancer cell survival and proliferation while modulating CYP3A4 activity could optimize the pharmacokinetics of chemotherapeutic agents, improving their efficacy. This combined approach might offer a more comprehensive strategy against cancer by addressing both the intrinsic proliferative capacity of cancer cells and external factors that influence drug response. Understanding the mechanisms by which CDK9 and CYP3A4 contribute to cancer development and progression is essential for the development of new therapeutic strategies. Further research is required to elucidate the molecular interactions involving these targets and to identify biomarkers that predict response to CDK9 and CYP3A4 inhibitors (Marra and Curigliano, 2019). CDK9 and CYP3A4 are critical components of the complex network underlying cancer biology, and their inhibition represents promising targets for therapeutic intervention. This approach has the potential to advance personalized medicine, enabling the development of more effective, tailored treatment regimens for cancer patients (Marra and Curigliano, 2019).

The crucial role played by Computer-Aided Drug Design (CADD) in the *in silico* molecular design to reduce the time required for drug conceptualization and to preserve the material, as demonstrated in recent significant works, is highlighted (Faris et al., 2024). In these studies, the emphasis is placed on guiding *in vitro* investigations through computer methods (Kapetanovic, 2008; Moroy and Tuffery, 2022; Nascimento et al., 2022; Al-Karmalawy et al., 2023). Various methods were employed in this research, leading us to design potent inhibitors against CDK9 and CYP3A4 enzymes.

In the first stage, the field-based 3D-QSAR method was used, starting with a collection of aligned ligands with known activity capable of inferring the impact of electrostatic, hydrophobic, and steric fields on biological activity or inactivity, field-Based QSAR swiftly transforms existing datasets into valuable QSAR models. Molecular docking was then used to obtain molecules exhibiting high interaction with the CDK9 and CYP3A4 enzymes, which may explain the strong response to the target. This step involved selecting the best candidates based on their affinity (kcal/mol). Subsequently, md and MM/PBSA, which are powerful *in silico* tools (Kukol, 2014), were employed to arrive at highly stable molecules. An ADMET study was conducted to understand the pharmacokinetic properties comprehensively. Additionally, predictive analysis of biological activity between active and inactive compounds was achieved using the Way2Drug portal.

## Results and discussion

### 3D-QSAR field-based analysis

The 3D-QSAR analysis, conducted using field-based tools in Schrödinger’s Maestro software, provides valuable insights. The preparation of input molecules aimed to establish a robust field-based model, guiding our understanding of the intricate relationship between pIC_50_ activity and influencing elements for enhancement. Through meticulous examination, four factors were identified for further exploration, guided by specific validation criteria outlined in Table 1.

**TABLE 1:** Performance metrics and statistical indicators for various factors in the study.

Factors	SD	R2	*R* ^2^_CV_	*R* ^2^ scramble	Stability	F	P	RMSE	Q^2^	Pearson-r
1	0.31	0.79	0.69	0.35	0.98	95.60	0	0.51	0.44	0.72
2	0.27	0.84	0.71	0.59	0.96	67.10	0	0.48	0.51	0.75
3	0.22	0.90	0.68	0.70	0.90	74.10	0	0.42	0.61	0.80
4	0.18	**0.94**	0.62	0.76	0.78	85.50	0	0.40	**0.66**	0.82

**FIGURE 1:**
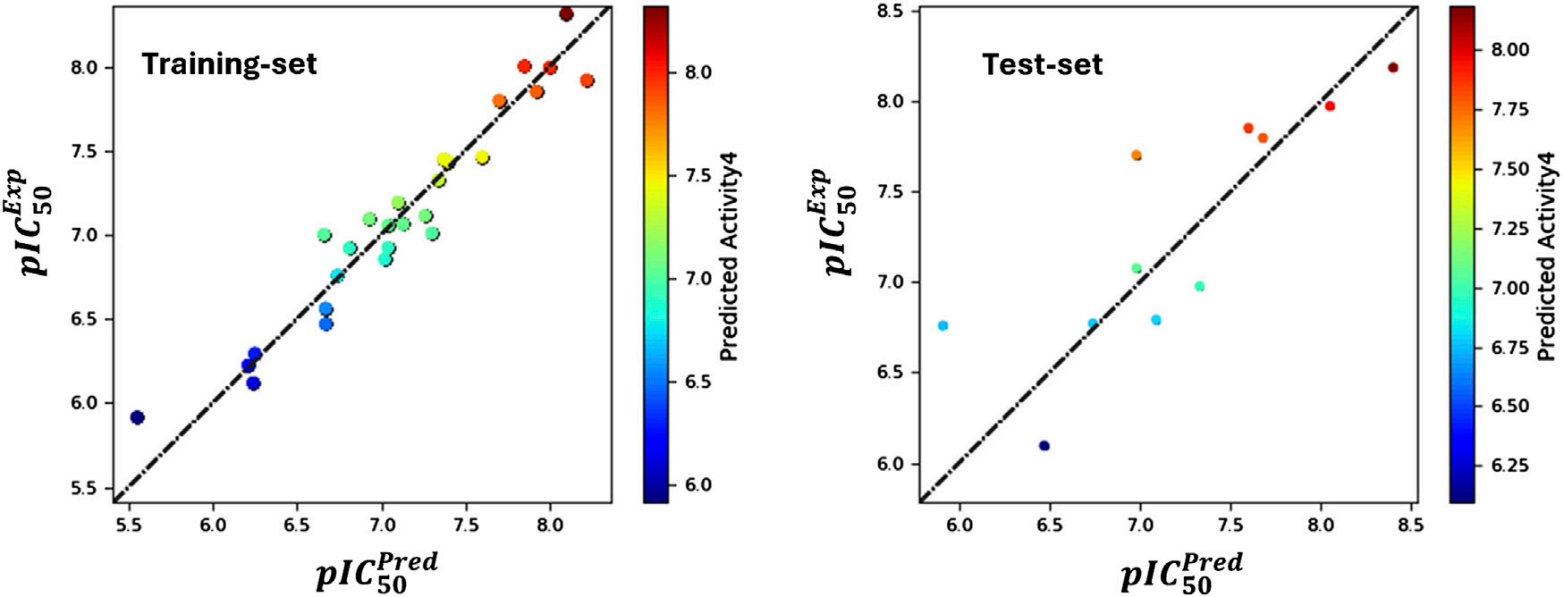
Contrasting the pIC_50_ values predicted by field-based methodologies for ' training and test sets with their corresponding actual values.

The analysis produced significant results, as shown in Table 1 and Figure 1. The model’s variability was reflected by a standard deviation (SD) of 0.18, while a high goodness of fit was demonstrated by an (*R* ^2^) value of 0.94, accounting for 94% of the variance in the data. This high *R* ^2^ value indicates a strong correlation between the independent and dependent variables. The 0.18 standard deviation (SD) illustrates how the model’s predictions vary from the mean. While a high SD suggests that data points are dispersed throughout a larger range, a low SD suggests that data points often tend to be near the mean. These metrics shed light on the significance of each predictor variable and the model’s overall predictive ability. The connection between the independent and dependent variables is shown graphically in Figure 1, often as a scatter plot superimposed on the regression line. This graphic aids in demonstrating how well the model fits the data points and identifies any patterns or trends. The strong R-squared value and low standard deviation show that the model can often explain data variability in the RAL L analysis. These findings imply that, based on the independent variables examined in the research, the model is a trustworthy resource for understanding or forecasting the dependent variable. *R* ^2^_(CV)_ obtained by applying cross-validation. This reflects the performance of the model outside the training sample, on new or unseen data. A value of 0.62 indicates that the model explains 62% of the variance in the data when predicting unseen compounds. Additionally, the model’s efficacy was highlighted by an *R* ^2^ value of 0.76 for the scrambled data. This value serves as a baseline for comparison, helping to confirm that the model is not merely fitting noise or random patterns in the data. A scrambled data *R* ^2^ close to that of the actual data might suggest potential overfitting, The stability value of 0.78 further supports the model’s robustness, indicating that it performs consistently across different subsets of the data. This value suggests that the model is not overly sensitive to changes in the data, which is a desirable characteristic in predictive modeling. The overall significance of the model was assessed through an F-statistic of 85.5, indicating statistical importance with analysis of the model performance parameters, including regression coefficients, *p*-values (optimal model with a *p*-value of 0), and confidence ranges, which are also given in Table 1. The Root Mean Square Error (RMSE) of 0.4 represented the average difference between observed and predicted values. According to the rule, a model with a Q^2^ value greater than 0.5 is considered reliable. A value of 0.66 indicates that the model explains 62% of the variance in the data when predicting unseen compounds, showcasing its reliability. A robust positive correlation between observed and predicted values, suggested by a Pearson correlation coefficient (Pearson-r) of 0.82, further reinforced the model’s validity.

### Analysis of contour maps

**FIGURE 2:**
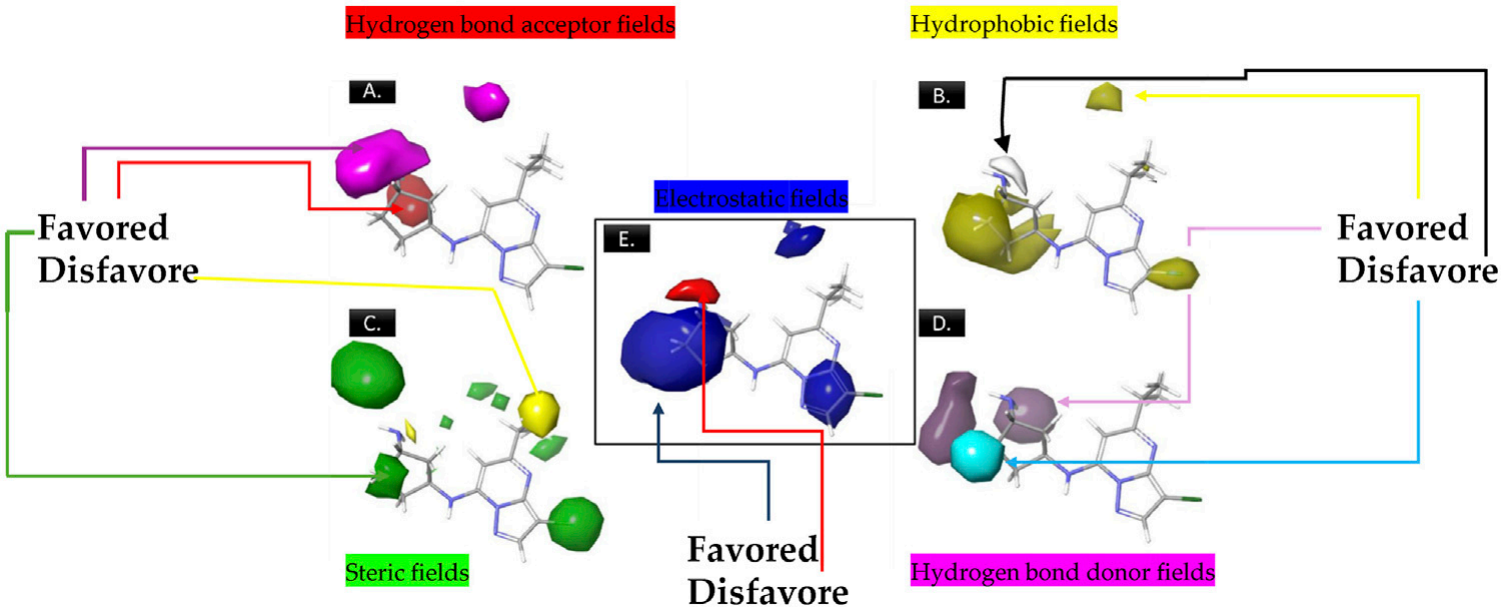
The contour maps generated for the compounds in the test set illustrate distinct fields, with specific colors indicating various characteristics. **(A)** Red signifies Gaussian hydrogen bond acceptor fields, indicating favored regions, while magenta denotes disfavored regions. **(B)** Yellow is employed for Gaussian hydrophobic fields to denote favored regions, while white signifies disfavored regions. **(C)** Green illustrates Gaussian steric fields, highlighting favored regions, and yellow indicates unfavorable regions. **(D)** Purple is used to depict Gaussian hydrogen bond donor fields for favored regions, while cyan represents disfavored regions. **(E)** Blue represents Gaussian electrostatic fields, showcasing favored electropositive regions and disfavored electronegative regions.

The analysis of contour maps for the best model four suggests the following key findings: In Figure 2A, red contours indicate an increase in biological activity within the cyclopentanamine group, attributed to acceptor groups in this region enhancing inhibitory activity. Conversely, magenta contours suggest a reduction in inhibitory activity, particularly in the hydrogen group of cyclopentanamine. In Figure 2B, yellow contours highlight the significance of chlorine in the cyclopentanamine group, emphasizing its role in CDK9 inhibition. The proximity of hydrophobic groups to propane further underscores their importance in this process. Figure 2C features green contours, underscoring the essential role of the chlorine atom, with steric effects near (1S,3S)-3-(12-azaneyl)cyclopentane-1-amine favoring increased inhibitory activity. In Figure 2D, purple contours predominate over cyan, indicating that donor groups in these regions contribute to increased inhibitory activity. Finally, Figure 2E shows blue contours on cyclopentanamine and 4-chloro-1H-pyrazole, suggesting electrostatic effects in these regions that favor an increase in biological activity.

Table 2 displays statistical analysis values for a Field-based model in 3D-QSAR. The factors considered are steric, electrostatic, hydrophobic, Hbonds acceptor, and Hbonds donor, each with corresponding numerical values: steric - 0.16 electrostatic - 0.18 hydrophobic - 0.28 Hbonds acceptor - 0.14 Hbonds donor - 0.24 these numerical values represent the contribution or importance of each factor in the 3D-QSAR model. Higher values indicate a greater impact of that specific factor on the model’s predictive capability. In this context, hydrophobic (0.28): This signifies a relatively significant role of hydrophobic interactions in the predictive power of the model. Varied hydrophobic characteristics in compounds likely result in substantial differences in their activities. Electrostatic (0.18): Indicates a contribution of electrostatic interactions to the model, albeit to a lesser extent compared to hydrophobic interactions. Hbonds Donor (0.24): Notes a notable impact of Hbonds Donor interactions on the model, suggesting that compounds with different abilities to donate hydrogen bonds exhibit distinct activities. Steric (0.16): Indicates a contribution of steric interactions to the model, with a relatively lower impact compared to other factors. Hbonds Acceptor (0.14): This suggests that Hbonds Acceptor interactions have the lowest impact among the considered factors in this model. These values offer insights into the relative importance of different molecular characteristics in influencing the biological activity predicted by the 3D-QSAR model.

**TABLE 2:** Statistical analysis of Field-based model in 3D-QSAR.

Factors	Steric	Electrostatic	Hydrophobic	Hbonds acceptor	Hbonds donor
1	0.17	0.14	0.33	0.06	0.30
2	0.19	0.14	0.29	0.08	0.29
3	0.12	0.16	0.28	0.14	0.30
4	0.16	0.18	0.28	0.14	0.24

### Designing novel compounds

**FIGURE 3:**
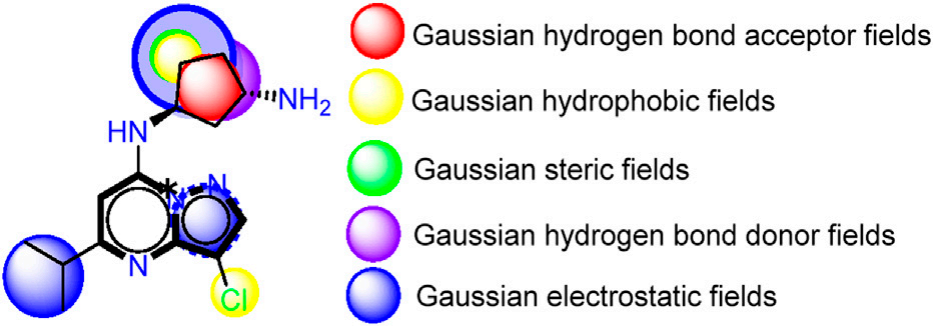
Guide for the design of new molecules based on a Field-based model; the best molecules were selected based on their affinity using the field-based model, and their predicted pIC_50_ values were utilized for the selection.

As a result of the analysis of the best model obtained from Model Four, for the field-based approach and the contour maps analysis, we obtain guidance that will facilitate the design of new inhibitors against CDK9 (Figure 3; Table 3). This leads us to design 15 molecules (See Supplementary Table S1), which will subsequently undergo molecular docking to select the best molecules with good affinity.

**TABLE 3:** The best new molecule designs using 3D-QSAR field-based methodology.

No	2D	pIC_50_(pred)	“Affinity in kcal/mol”
T1	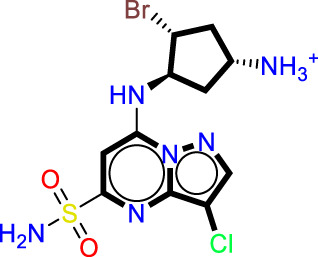	8.59	−8.81
T2	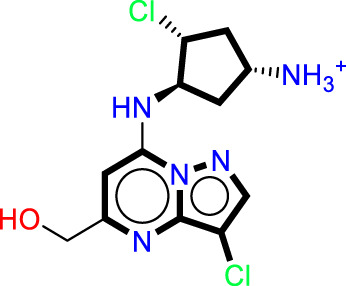	7.24	−8.78
T3	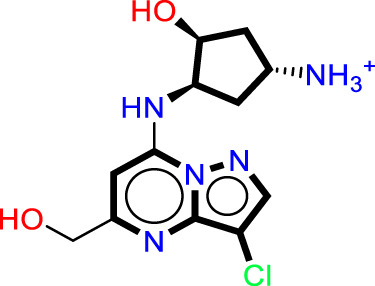	8.043	−8.68
Reference	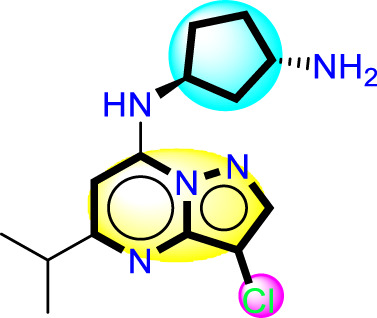	8.40	−8.40

The statistical results from Model four provide insight into the relative importance of various factors influencing the biological activity of this molecule. The steric factor, as outlined in Table X, pertains to the size and shape of the molecule. With its aromatic rings and carbon chain, this molecule exhibits structural rigidity, which can influence its interactions with other molecules or biological targets. Regarding electrostatics, the reference structure indicates that the quaternary ammonium group imparts a positive charge to the molecule, while the carbonyl and hydroxyl groups can engage in electrostatic interactions, including hydrogen bonding. These electrostatic properties are critical for molecular interactions and target recognition. In terms of hydrophobicity, the aromatic portions of the molecule contribute to its hydrophobic nature. The aromatic rings and the chlorine group, primarily composed of nonpolar carbon atoms, promote hydrophobic interactions with similar environments. The oxygen atoms in the hydroxyl and carbonyl groups can act as hydrogen bond acceptors, forming bonds with suitable donors, which can stabilize molecular complexes and influence the molecule’s affinity for biological targets. Although less pronounced, the molecule can also function as a hydrogen bond donor, primarily through the quaternary ammonium group, though this capacity is limited by the molecule’s positive charge. Model four highlights the relative importance of each factor in determining the biological activity of this molecule. In this specific case, electrostatic and hydrophobic interactions appear to play a more decisive role than hydrogen bonding interactions. This insight could guide the modification of the molecular structure to optimize the desired activity by adjusting steric, electrostatic, and hydrophobic properties while considering hydrogen bond acceptor and donor capacities.

## Molecular docking investigation

### Molecular docking for CDK9 exploration

**FIGURE 4:**
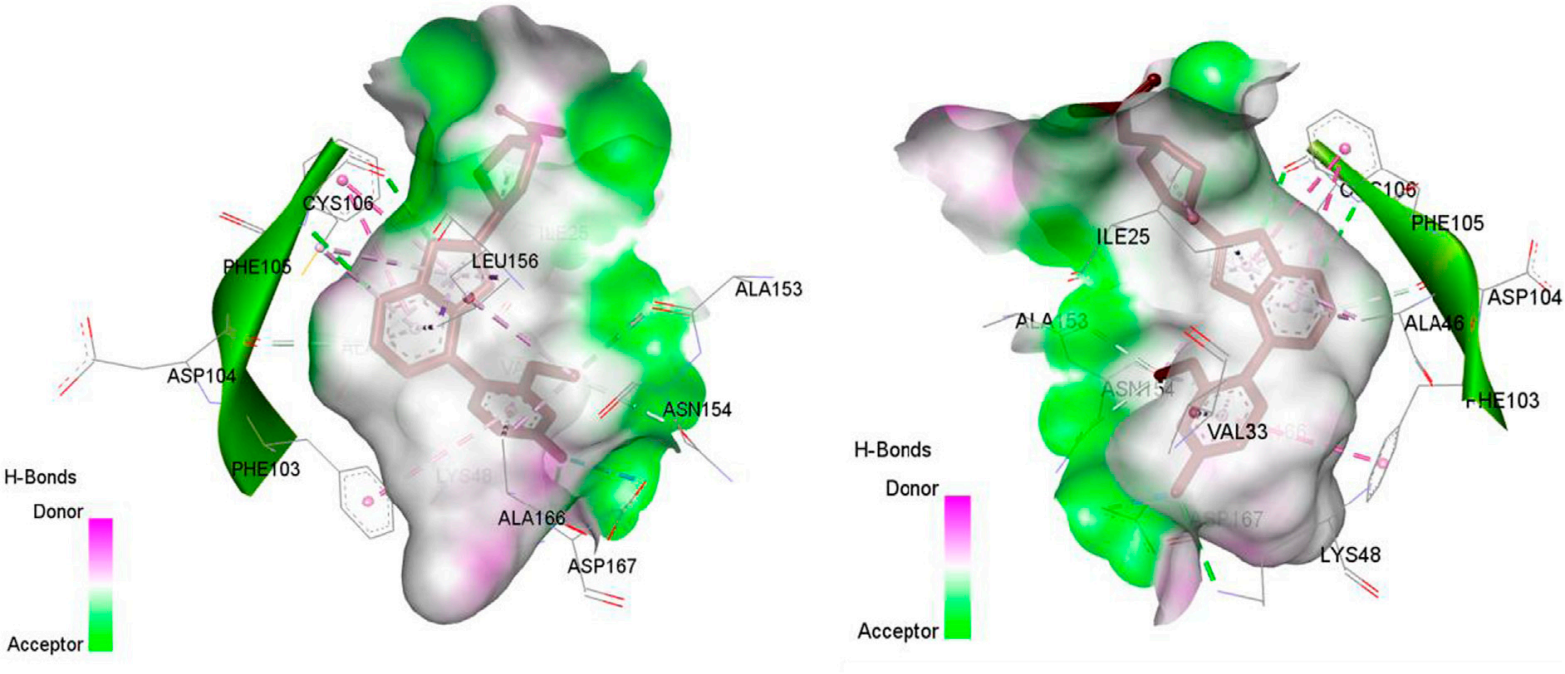
The representation of co-crystallized ligand in the active site of CDK9, the target with PDB: 7nwk.

**FIGURE 5:**
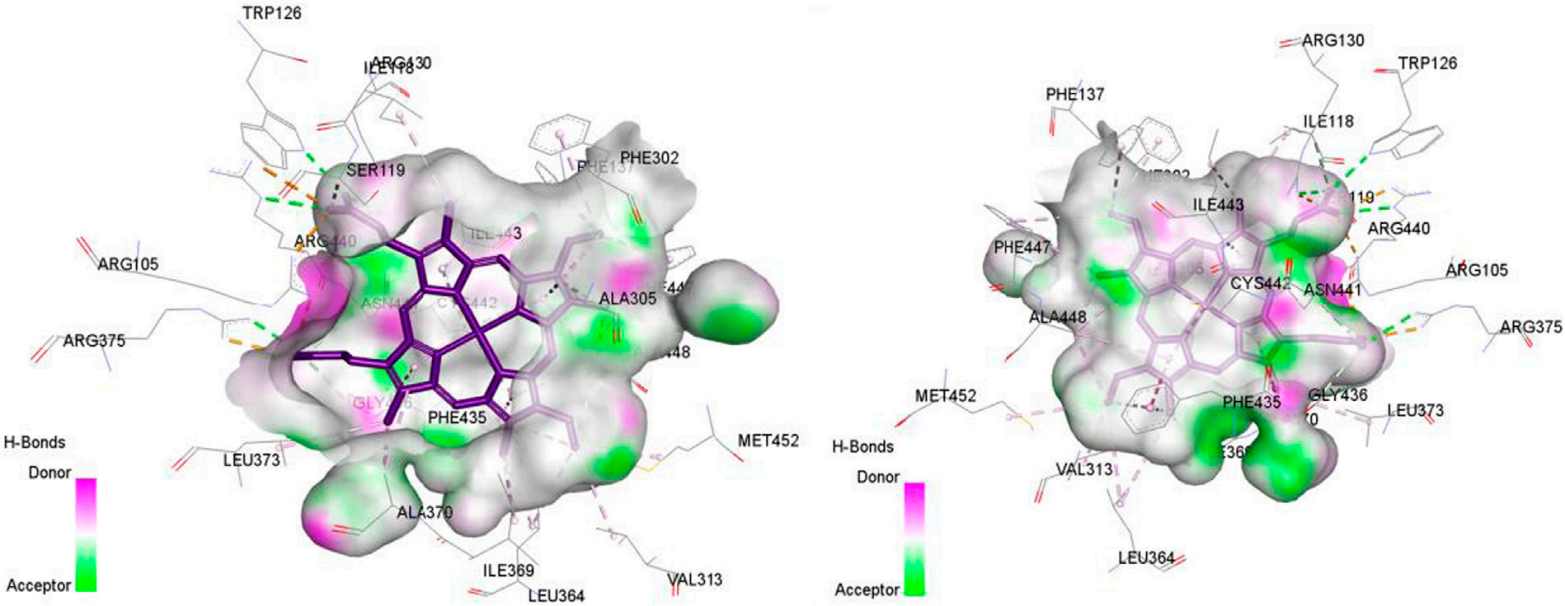
The representation of HEM (Heme is a complex formed by a porphyrin ring (which contains carbon, hydrogen, and nitrogen) with an iron atom at its center.) in the active site of CYP3A4, the target with PDB: 8wes.

In molecular docking, the identification of active sites remains a key factor for target inhibition. In our study, for the determination of active sites, we relied on the coordinates of co-crystallized ligands to accurately define them, as these are based on previous experimental data and precise biological analyses, as shown in Figures 4, 5.

**FIGURE 6:**
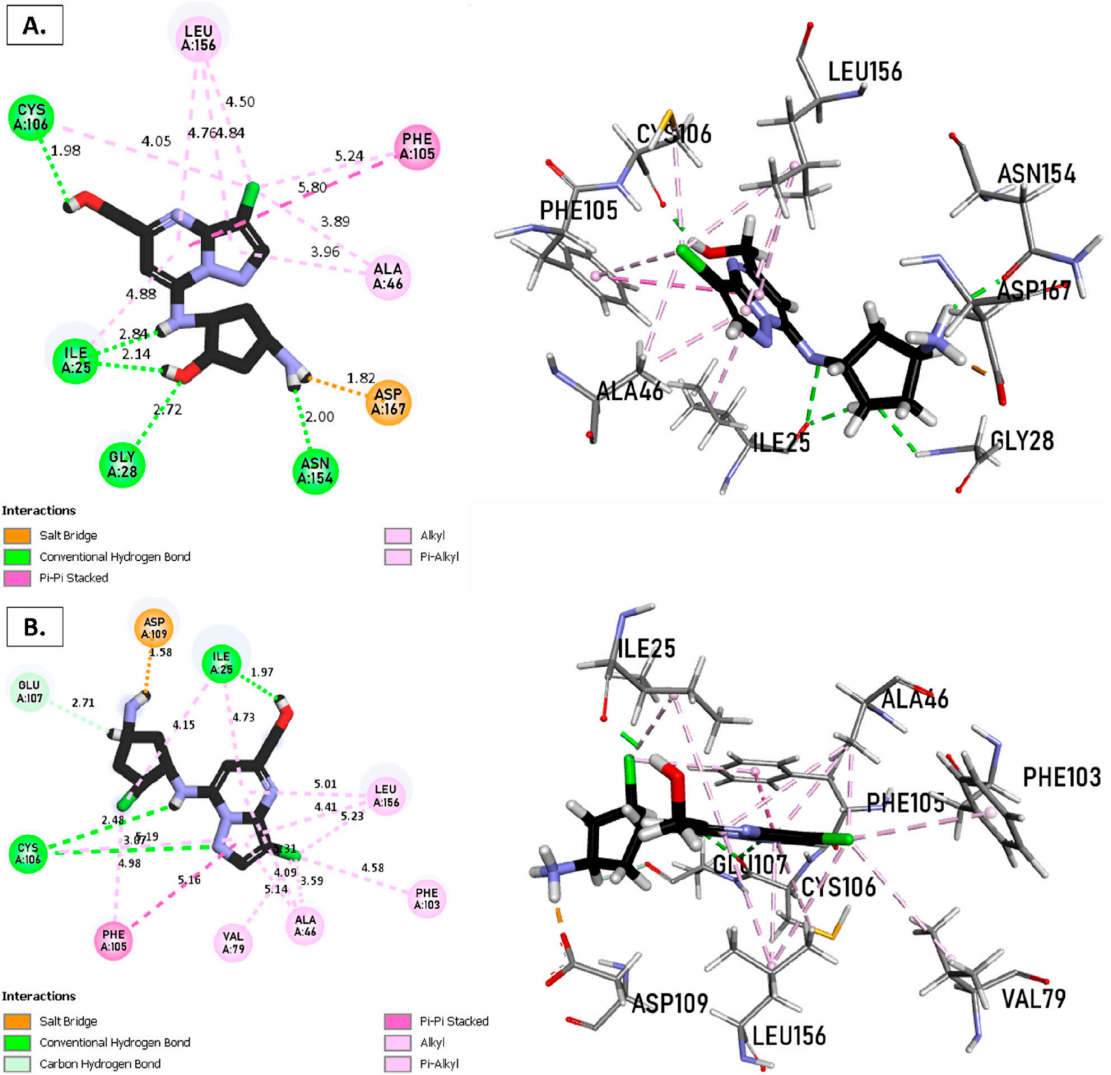
Non-covalent binding interactions of novel compounds **(A)** (T1) and **(B)** (T2) with CDK9 (PDB ID: 7NWK): 3D and 2D diagram illustrations.

**FIGURE 7:**
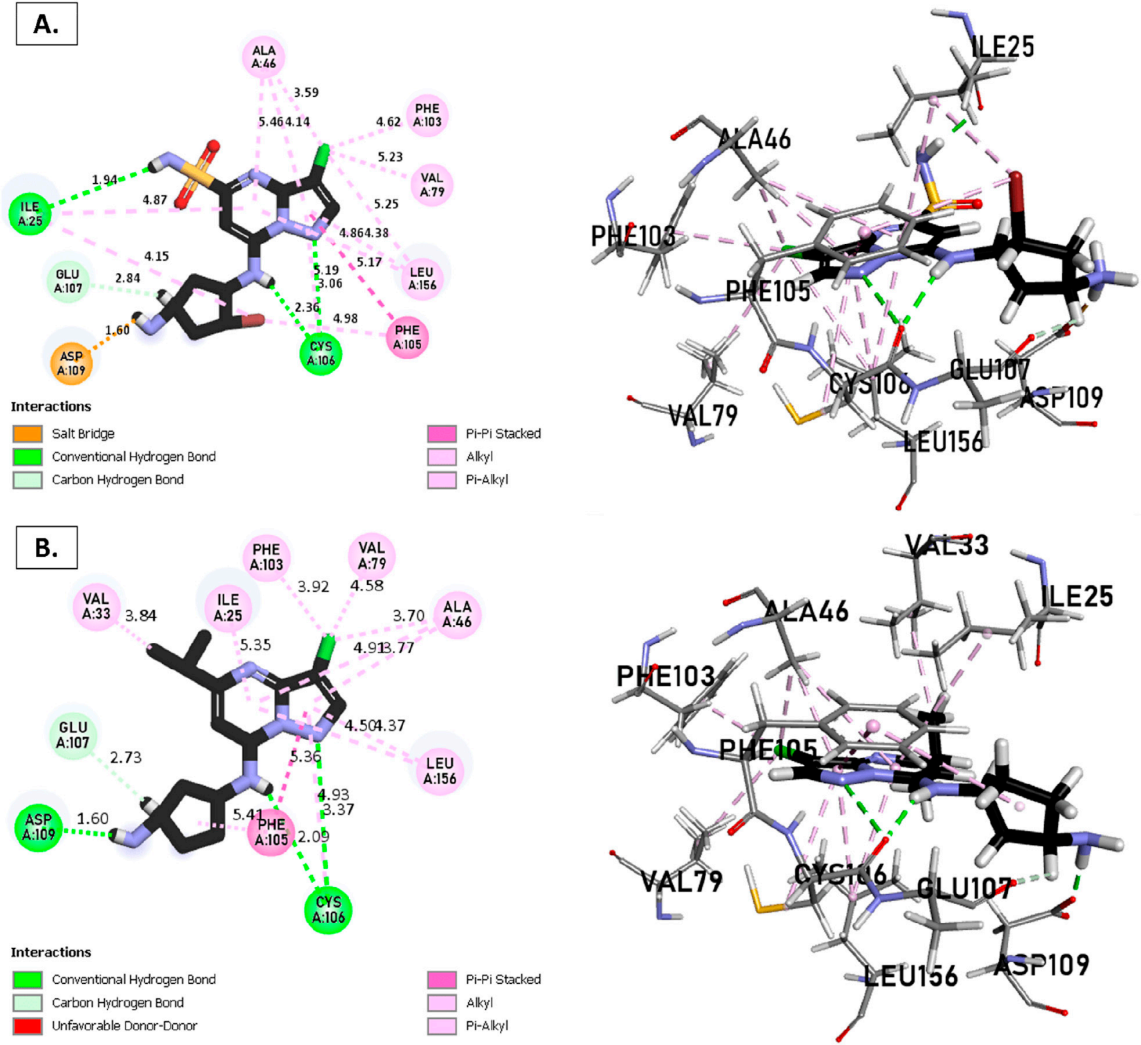
Non-covalent binding interactions of novel compounds **(A)** (T3) and **(B)** (Reference) with CDK9 (PDB ID: 7NWK): 3D and 2D diagram illustrations.

The analysis of molecular docking results for the new molecules with the CDK9 protein suggests the following: Compound T1 (Figure 6) presents five hydrogen bonds with Cys106, Ile25, Gly28, Asn154, and a salt-bridge bond with Asp167. It also exhibits alkyl and pi-alkyl bonds and pi-pi stacked interactions with various residues, including Leu156, Phe105, and Ala46. Next, Compound T2 (Figure 6) features three hydrogen bonds with Cys106 and Ile26, various pi-alkyl and alkyl bonds, pi-pi stacked interactions with residues such as Phe105, Val79, Ala66, Fhe103, Leu156, Ile25, and a salt bridge with Asp109. Additionally, Compound T3 (Figure 7) shows three hydrogen bonds with Cys106 and Ile25, along with various alkyl, pi-alkyl, and pi-pi stacked interactions with residues like Ala46, Phe103, Val79, Leu156, Phe105, and a salt bridge with Asp109. Finally, the reference compound (Figure 7) exhibits three hydrogen bonds with Asp109 and Cys106, along with various pi-alkyl and alkyl bonds, pi-pi stacked interactions with residues Val33, Ile25, Phe103, Val79, Ala46, Leu156, and Phe105.

Upon analyzing the docking results, it is evident that for molecules T1, T2, and the reference, the predominance of hydrogen bonds occurs at the pyrazolopyrimidine and diazaneyl)methyl)cyclopentanone groups, and for molecule T2, at the -OH groups. Molecule T1 is in the SO_2_-NH group, and for the reference molecule, it is in the propane group. This suggests that replacing propane with a donor or acceptor group favors the creation of hydrogen bonds. Despite the absence of a hydrogen bond at the pyrazolopyrimidine groups for molecule T3, the presence of hydrogen bonds at the diazaneyl)methyl) cyclopentanone group suggests the significant presence of hydrogen bonds facilitated by newly substituted groups. Furthermore, in comparison to the reference, the presence of a hydrogen bond at the -OH groups is indicated.

The docking results help explain the good non-covalent interactions for the new compounds with the CDK9 protein.

### Docking molecular for CYP3A4

**FIGURE 8:**
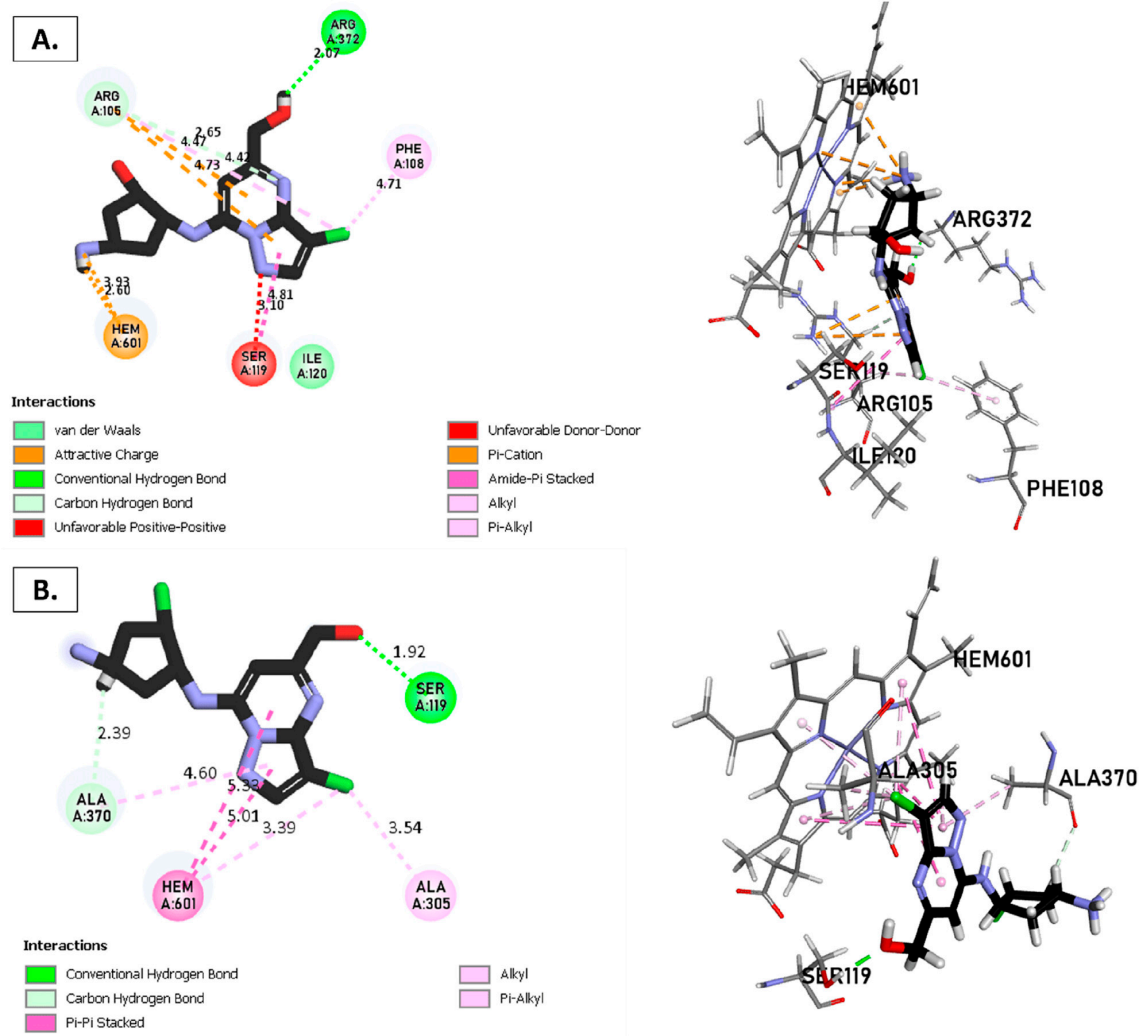
Non-covalent binding interactions of novel compounds: **(A)** T1 and **(B)** T2 with CYP3A4 (PDB ID: 8EWS), illustrated in 3D and 2D diagrams.

**FIGURE 9:**
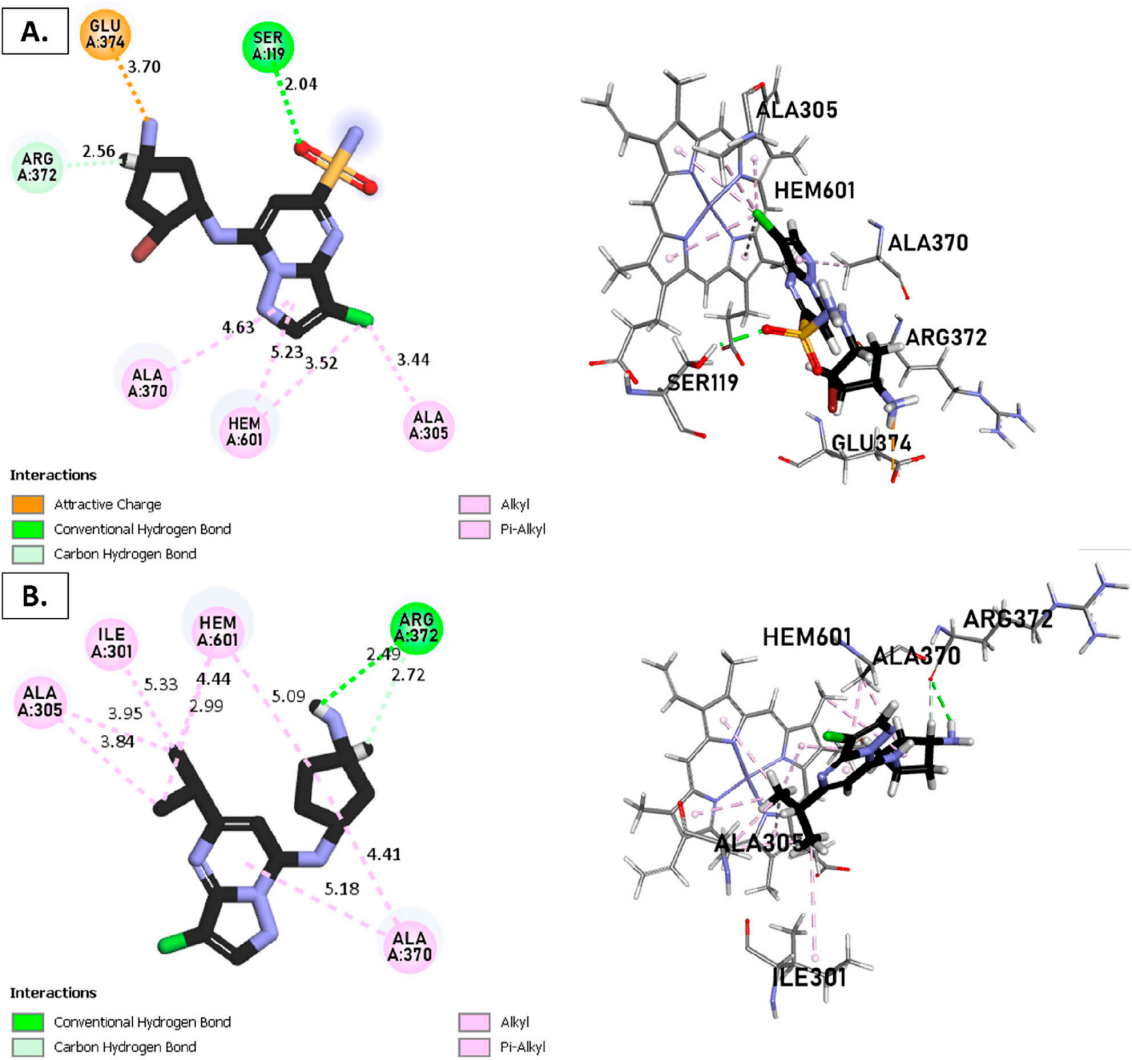
Non-covalent binding interactions of novel compounds: **(A)** T1 and **(B)** T2 with CYP3A4 (PDB ID: 8EWS), illustrated in 3D and 2D diagrams.

The metabolism of the enzyme CYP3A4, involving compounds T1, T2, T3, and reference, was investigated (Figures 8, 9). The CYP3A4 (***ID***: 8EWS) selected for this study also exhibits a high resolution and is a good fit for experimental data through the better ligand structure. This protein choice provides valuable insights into the metabolic processes within the enzyme. Notably, a co-ligand, HEM, remains a target of interest due to its ability to activate CYP3A4. Consequently, there is a focus on inhibiting this enzyme as a potential strategy to modulate its activity.

Molecular docking analysis with the CYP3A4 enzyme and new compounds suggests the following: Molecule T1 exhibits non-covalent interactions, including a hydrogen bond with Arg372, an alkyl bond with Phe108, four pi-alkyl bonds, attractive charge, and van der Waals interactions with Arg105, and two pi-cation bonds with HEM601. Next, for molecule T2, non-covalent interactions are observed, including a hydrogen bond with Ser119, an alkyl bond with Ala305, three pi-alkyl bonds, pi-pi stacked interactions with HEM601, and two pi-alkyl and carbon-hydrogen bonds with Ala370. Additionally, for molecule T3, non-covalent interactions include a hydrogen bond with Ser119, an alkyl bond with Ala305, two pi-alkyl bonds with HEM601, and one pi-alkyl bond with Ala370. There is also a carbon-hydrogen bond with Arg372 and an attractive charge interaction with Glu374. Finally, the reference molecule represents non-covalent interactions, including a hydrogen bond and a carbon-hydrogen bond with Arg372, two alkyl bonds with Ala305, one alkyl bond with Ile301, and two bonds with HEM601 involving pi-alkyl and alkyl interactions. Additionally, there are two pi-alkyl bonds with Ala37.

The most crucial aspect in the molecular docking analysis for the new compounds with CYP3A4 is to have interactions with HEM601, which plays a crucial role as an essential cofactor. Heme is a complex organic molecule containing a porphyrin nucleus linked to a ferrous ion (Fe^2+^). In the context of CYP3A4, heme is associated with the protein that forms a heme-protein structure. The CYP3A4 vital contains a heme molecule connected to an iron atom. This iron-heme bond allows cytochromes P450 to perform the oxidation-reduction processes necessary for drug metabolism. Without heme iron, CYP3A4 could not carry out its metabolic job. That is why the target molecules were selected for this position. Compounds that act as inhibitors for CYP3A4 are those exhibiting good interactions with HEM601 (a chemical substance that is not a protein instead is necessary for a protein, frequently an enzyme, to function biologically. Cofactors can be thought of as helpers for biochemical transformations because they are commonly engaged in catalytic processes), as observed particularly in the case of molecule T2, followed by molecule T3 and the reference compound. These interactions with HEM601 are important as they may influence the efficiency and specificity of enzyme inhibition by these compounds. Thus, the presence of strong interactions with HEM601 can serve as an indicator of the potential effectiveness of the compounds as CYP3A4 inhibitors.

## Molecular Dynamic’s

### RMSD, RMSF, Rg, SASA and DSSP analyses

The RMSD, RMSF, Rg, solvent accessible surface area (SASA), FEL, and PCA studies offer important insights into several facets of the complexes under study. The average deviations in atomic locations between the initial and final structures are measured by RMSD, which makes it possible to evaluate the complexes’ stability and conformational changes. By measuring the average changes in atomic locations throughout the simulation, RMSF reveals the relative stability and flexibility of residues. Rg calculates a molecule’s three-dimensional compactness, revealing details on the dimensions and form of the complex under study. SASA assesses a molecule’s solvent-accessible surface to provide information about residue accessibility and environmental exposure. FEL assesses a molecule’s structural flexibility by measuring its capacity to deform or alter shape. PCA analysis is a statistical method that identifies the primary modes of variation in the structures under study, therefore reducing the complexity of the data. By enabling the evaluation of the investigated complexes’ interactions, stability, flexibility, compactness, solvent accessibility, and variation modes, these metrics provide information that improves our comprehension of their structural behavior and characteristics.

The analyses of RMSD, RMSF, Rg, and SASA in Supplementary Figure S1 provide the following results: The RMSD analyses for the compounds show a favorable stability ranging between 1 and 3 Å during the 100 ns simulation. Analysis of Rg and SASA of the novel design compound compared to the reference with SASA (2,300 Å^2^) and Rg (22.5 Å) suggests the compactness of all new compounds with CDK9 tend to have the same compactness as compound T1, which shows a good compactness with a Rg and SASA of 22.56 Å and 2,300 Å^2^ respectively, unlike the others, which show little variation. Fortunately, as the results suggest, the Rg and SASA of all complexes indicate stability without exhibiting excessively high peaks.

The results of RMSD (Supplementary Figure S2) for new ligands with CYP3A4 It has been noted that it exhibits remarkable stability and yields satisfactory results for those who calculate RMSD. The T1, T2, and T3 RMSDs fall between 1 and 2 Å compared to the reference. Similarly, the Rg and SASA (Supplementary Figure S2) studies show a good understanding of the ligand-protein structures, with the Rg queue highlighting this understanding at 50 ns. The analyzed Hbonds clearly show how important it is to have enough Hbonds (Supplementary Figure S2) during simulation without occupation, which validates the target’s docking results. With a minimum of one and a maximum of up to 5, the hydrogen bonding results for the novel compounds with the target indicate good results. This suggests the existence of hydrogen bonds and highlights their significance for the stability of the resulting complexes. For the results of the RMSF (Supplementary Figure S3) analysis indicate the stability of the complexes concerning the residues during the simulation, exhibiting moderate flexibility.

Throughout the simulation, an analysis was carried out using the defined secondary structure of proteins (DSSP) (Supplementary Figures S4, S5) to look at secondary structural elements such as alpha helices, beta sheets, and coils. Multiple time intervals were used to acquire the trajectories. The DSSP analysis for the new molecules over 100 ns reveals a clear change from Turn to Coil starting at 530 ns for residue SER7, based on the reference of new compounds, compound T1, interacting with the protein. In complexes T2 and T3, which interact with the protein, reagent LYS56 undergoes a transition from 0 ns. These findings suggest that the protein may experience significant conformational changes in its interaction with the previously described chemicals over time.

**FIGURE 10:**
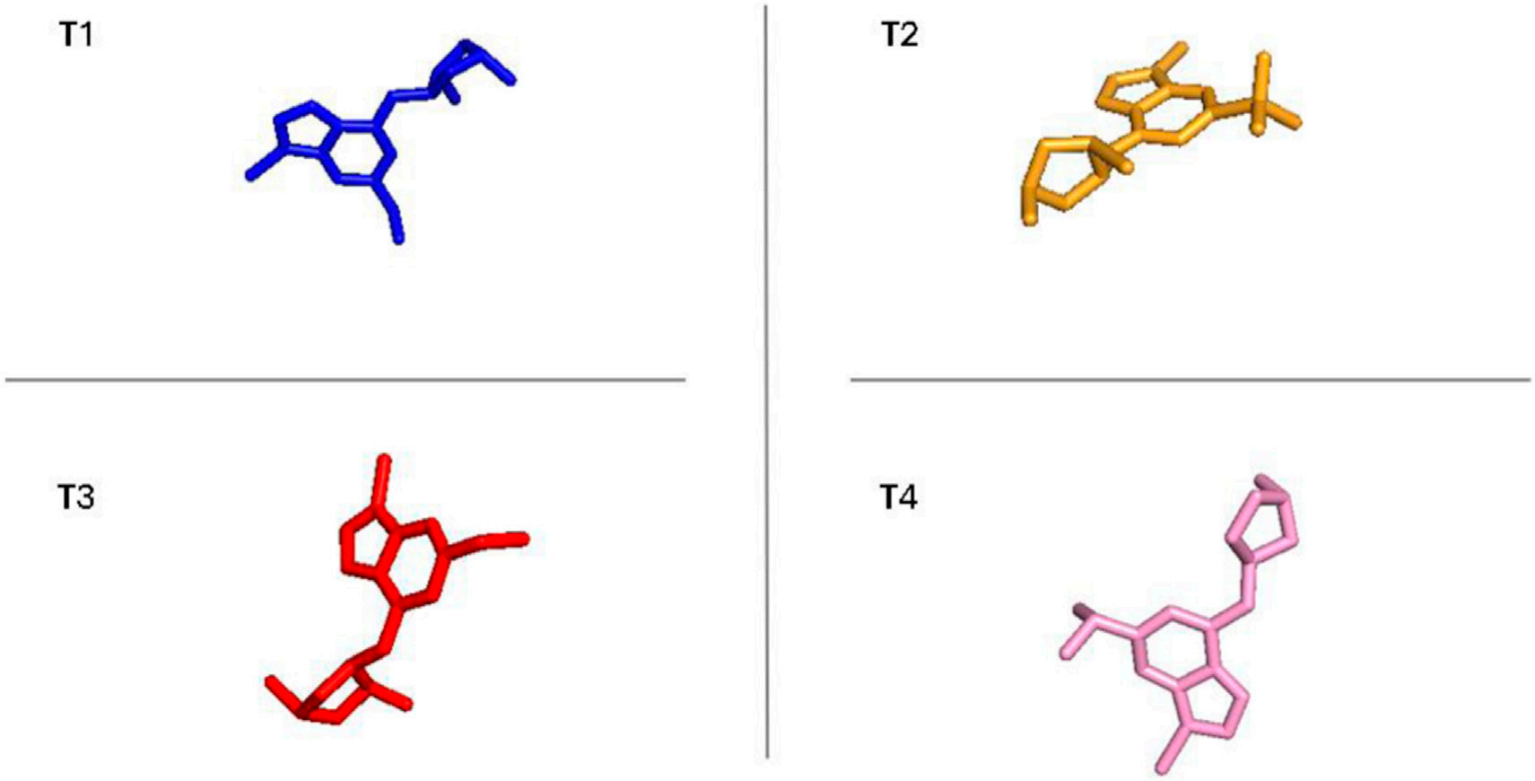
The most stable conformations of the new compounds and ref with CYP3A4 corresponding to the energy minima for the new inhibitors.

The outcomes presented in Supplementary Figures S4–S8, and S9 reveal the following from PCA and FEL analyses. In terms of PCA results, T1 exhibited PC1 and PC2 values ranging from −2 to −3, respectively. T2 displayed PCA values for PC1 and PC2 within the ranges of −2 to −2 and two to −4, respectively. T3 showcased PCA results for PC1 and PC2, spanning from −2 to −2 and −2 to −3, respectively. The reference compound demonstrated PCA values for PC1 and PC2 ranging from −2 to −2 and −2 to −3, respectively. Moving on to FEL, T1 displayed a stable conformation energy minimum within an RMSD range of 0.3 nm and an Rg range between 2.04 and 2.06 nm T2 exhibited a stable conformation energy minimum within an RMSD of 0.34 nm and an Rg of 2.04 nm T3 demonstrated stable conformation energy minima located between an RMSD of 0.25 nm and an Rg of 2.04. The reference compound showed stable conformation energy minima within an RMSD of 0.2 nm and an Rg of 2.02 nm. Similarly, using the same methodology as the earlier findings shown in Figure 10 supply the conformational analysis results for the most stable energies in the FEL study of the novel compounds, including CYP3A4.

## Free binding energy (MM/PBSA)

The findings from MM/PBSA analysis serve as a basis for prioritizing compounds for additional optimization or steering the development of new ligands with enhanced binding affinity (Table 4). This analytical approach proves invaluable in comprehending and foreseeing the binding energetics of molecular complexes, thereby assisting in the design and optimization of drugs based on the structure. The MM/PBSA analysis results, presented in Table 4 unveil the energy contributions of various components and complexes. The van der Waals energy values (ΔVDWAALS) indicate non-covalent interactions (dispersion) between the ligand and protein. The energies are slightly weaker for T1-CDK9 and T2-CDK9 compared to T3-CDK9 and Ref-CDK9, suggesting weaker van der Waals interactions for T1 and T2. The polar solvation energy (ΔEPB) represents the solvent’s polar effect on the complex. T3-CDK9 has the highest polar solvation energy, which could compensate for the strong electrostatic interactions observed. The total solvation energy (ΔGSOLV) represents the sum of gas-phase energies and solvation energies. T1-CDK9 has the most negative total energy, indicating the strongest overall interaction with CDK9, closely followed by T2-CDK9, then T3-CDK9, and finally Ref-CDK9. For global energy, T1-CDK9 has the most favorable interaction energy, making it the best candidate in terms of complex stability. T2-CDK9 is very similar to T1-CDK9 but slightly less favorable. T3-CDK9 has very strong electrostatic interactions and high solvation energy, reducing its total energy despite strong gas-phase interaction. Ref-CDK9 has the least favorable total energy, indicating weaker interactions with CDK9 compared to the other complexes.

**TABLE 4:** Energy contributions and binding characteristics of complexes.

Energy (kcal/mol)	T1-CDK9	T2-CDK9	T3-CDK9	Ref-CDK9
Δ_VDWAALS_	−2.97	−3.61	−12.9	−13.21
ΔE_EL_	−104.6	−105.93	−186.78	−74.29
ΔE_PB_	94.53	96.98	192.29	81.23
ΔG_GAS_	−107.57	−109.54	−199.68	−87.50
ΔG_SOLV_	92.77	95.18	189.02	79.53
Δ_TOTAL_	−14.8	−14.35	−10.66	−7.98

## ADME-Tox analysis

Under ADMET rules (Pires et al., 2015; Xiong et al., 2021; Faris et al., 2024c), for designing new compounds (Tables 5, 6), the value of logS reflects the drug’s solubility. The smaller the value, the less soluble the compound is in water. When logS are less than −6, the compounds are considered poorly soluble and insoluble. A molecule with less than 30% absorption is considered weakly absorbed, while molecules with an absorption greater than 30% are considered to have high absorption. The unit of BBB penetration is cm/s. Molecules with logBB greater than −1 are classified as BBB^+^ (Category 1), while molecules with logBB less than or equal to −1 are classified as BBB^−^ (Category 0). BBB^−^ indicates that the molecule has a low capacity to penetrate the blood-brain barrier (BBB) or does not penetrate at all. This may be desirable for certain drugs targeting the central nervous system (CNS) to minimize side effects or undesirable interactions with the brain. BBB^+^ indicates that the molecule has a high capacity to penetrate the BBB. This may be desirable for certain drugs that require direct access to the brain to be effective in treating CNS diseases.

**TABLE 5:** Pharmacological profiling of compounds T1, T2, T3, and reference: Assessing permeability, absorption, enzyme interactions, and toxicity.

Compound		T1	T2	T3	Reference
Caco2 permeability		0.18	0.74	−0.05	1
Intestinal absorption (human)		68	76	78	93
substrate	CYP2D6	No	No	No	No
	CYP3A4	No	No	No	Yes
inhibitor	CYP1A2	No	No	No	Yes
	CYP2C19	No	No	No	No
	CYP2C9	No	No	No	No
	CYP2D6	No	No	No	No
	CYP3A4	No	Yes	No	No
Total Clearance		0.44	1.22	1.19	0.82
AMES toxicity		No	Yes	No	No

**TABLE 6:** Physicochemical characterization of compounds T1, T2, T3, and reference: Evaluating molecular weight, lipophilicity, and structural features.

Compound	Rule	T1	T2	T3	Ref
MOL_WEIGHT	100–600	410.705	317.2	298	293
LogP	0 to 3 log mol/L	−0.021	0.66	−0.58	2.79
ROTATABLE_BONDS	0–11	3	3	3	3
ACCEPTORS	0–12	6	5	6	5
DONORS	0–7	3	3	4	2
SURFACE_AREA	0–140	139	125	120	123

**FIGURE 11:**
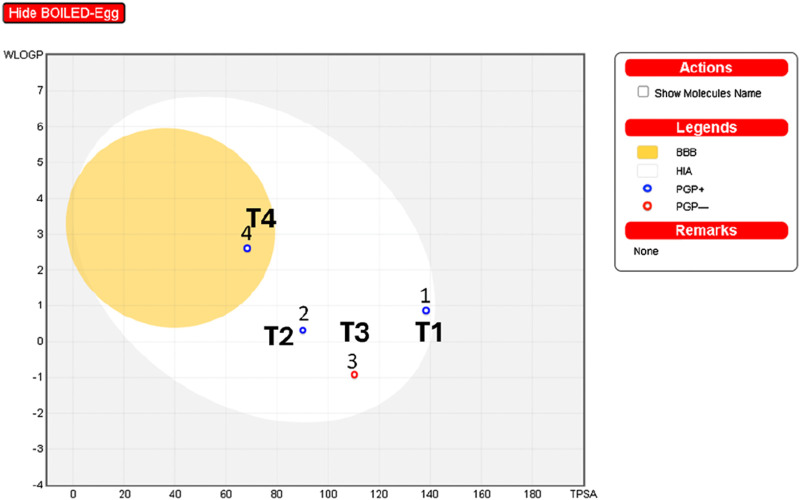
Analysis of boiled egg using SwissADME: T1, T2, T3, and reference.

The interpretation of the SwissADME results suggests the following: The analysis of boiled egg predicts (Figure 11) that molecule four is in the yellow zone of the blood-brain barrier (BBB), indicating potential permeability, while the rest of the molecules (1, 2, and 3) are situated in the white zone of high absorption (HA). Additionally, molecules 1, 2, and four are identified as P-glycoprotein substrates (PGP+), implying that they may interact with P-glycoprotein. On the other hand, molecule three is classified as a P-glycoprotein non-substrate (PGP-), suggesting a different interaction profile with P-glycoprotein compared to the other compounds.

ADMET analysis indicates that Compound T1 exhibits the lowest forecasted Caco-2 cell permeability and human intestinal absorption, potentially limiting its oral bioavailability. Except for REF, all compounds are projected to be non-substrates for major drug-metabolizing CYP enzymes like CYP2D6 and CYP3A4, while REF’s clearance might be elevated due to its CYP3A4 substrate status. Only reference is anticipated to inhibit CYP1A2 and CYP3A4 activities, raising concerns about potential drug-drug interactions. Total clearance values suggest that Compound T2 may have the shortest half-life, whereas 18 and REF could have longer durations of action. Compound T2 is predicted to be positive in the AMES toxicity assay, indicating potential mutagenic effects, while the other compounds are expected to be non-mutagenic. Reference demonstrates undesirable interactions with drug-metabolizing enzymes, posing potential challenges to its efficacy or safety. T3 and T1 display better ADMET profiles, although they may have lower oral bioavailability than 33 based on permeability/absorption predictions. Overall, Compound T3 appears to strike the most favorable balance of properties.

## Drug-Liknes

The analysis of physicochemical properties indicates that all compounds possess molecular weights falling within the range of 100–600 g/mol, as specified by Rule 1. T2 and 39 exhibit LOGP values within the 0–3 log mol/L range, aligning with Rule 2. Conversely, T3 and REF deviate from this range. All compounds feature between 0-11 rotatable bonds, meeting the criteria of Rule 3. T2, 18, and REF demonstrate hydrogen bond acceptor counts within the 0-12 range defined by Rule 4. However, Compound 39 surpasses this limit with 13 acceptors. T2 and 18 have hydrogen bond donor counts within the 0-7 range, by Rule 5. On the contrary, T1 and REF fall outside this specified range. The surface areas of all compounds fall between 0–140 A^2^, as per Rule 6. T2 and T1 adhere to some but not all rules. Compound 33 satisfies most rules, making it a potentially acceptable candidate. T3 and REF violate multiple rules, making them less desirable for the design of new molecules.

ADMET analysis reveals distinct physicochemical properties among the compounds. Compound T3 emerges as a potential candidate, meeting more criteria and displaying a balanced ADMET profile. T3 and Ref, while violating multiple rules, may present challenges in designing new molecules. Consideration of these factors aids in identifying promising drug candidates.

## Activity biology

The Way2Drug web server was utilized to determine the probability of activity for newly designed molecules against CDK9 (Table 7). The results indicate that Compound T1 exhibits a Pa (probability of being active) of 0.344 and a Pi (probability of being inactive) of 0.081, classifying it as a CDK9/cyclin inhibitor. T2 and T3, along with the Ref, have Pa values of 0.444, 0.458, and 0.452, respectively, and Pi values of 0.028, 0.024, and 0.026, respectively. The specific activity for T2 and T3, as well as the reference, is not provided.

**TABLE 7:** Probability assessment of CDK9/Cyclin T1 inhibition for designed compounds.

Compound	Pa	Pi	Activity
T1	0.458	0.024	CDK9/cyclin T1 inhibitor
T2	0.444	0.028	
T3	0.344	0.081	
Ref	0.452	0.026	

The results suggest potential activity for the designed molecules, and further interpretation is encouraged based on these probabilities and associated activities.

From the CADD analysis, T1-CDK9 exhibits strong binding interactions with CDK9, indicated by a notably negative total energy of −14.8 kcal/mol, which suggests enhanced complex stability. However, its van der Waals interactions are weaker compared to other compounds. Despite its strong binding affinity, T1-CDK9 encounters pharmacokinetic limitations, including low predicted Caco-2 cell permeability and moderate human intestinal absorption (68%), which could hinder its oral bioavailability. Although it does not interact with major drug-metabolizing enzymes, which is a positive feature, its overall drug-likeness profile indicates it may not fully meet the criteria for an ideal drug candidate.

## Conclusion

In conclusion, this study has effectively utilized a computer-assisted design approach to identify potential inhibitors for CDK9 and CYP3A4 proteins. By employing a predictive QSAR model and *in silico* synthesis, the study has identified several promising compounds. The new compounds (T1, T2, T3) showed encouraging results for their potential as inhibitors of CDK9 and CYP3A4 proteins in molecular docking, molecular dynamics, and ADMET analyses when compared with a reference compound. According to molecular docking, these compounds exhibit considerable and stable non-covalent interactions with both target proteins. Of particular note are strong hydrogen bonding and pi-alkyl interactions, which are critical for the compounds’ binding affinity. The stability of these interactions was verified by molecular dynamics simulations, wherein the compounds demonstrated favorable RMSD, RMSF, Rg, and SASA values, signifying strong structural stability and compactness during the simulations. Furthermore, according to the MM/PBSA analysis, T1 and T2 have the best binding energies, suggesting that they have a great potential for interaction with CDK9. T3 also showed promising but marginally weaker interactions. The drugs usually have favorable pharmacokinetic features, according to ADMET profiling, with T3 having the best balance of toxicity profiles, enzyme interaction, and absorption. In contrast to the reference, T1 and T3 can have reduced oral bioavailability. Notably, T2 displayed possible mutagenic properties that may restrict its application in medicine. With its good binding affinity, stability, and ADMET qualities, compound T3 stands out as the most drug-like candidate overall. This makes it a viable contender for further development and optimization as a therapeutic agent targeting CYP3A4 and CDK9. Methods and Materials.

### Dataset

In this study, a series of new molecules, KB-0742, a potent, selective, orally bioavailable small molecule inhibitor of CDK9 for MYC-dependent cancers, was relied upon (Freeman et al., 2023). These molecules have not yet been treated using *in silico* methods to test the best molecules in this series. Based on it, powerful new molecules can be produced. The series comprises 39 molecules with their IC_50_ (nm) activity treated at pIC_50_ based on the logarithm -log(10*-9*IC_50_(nm)). These molecules were divided into a training set of 28 molecules and a test set of 11 molecules (Supplementary Table S1), with the most active molecule in the training set to ensure good predictivity of the activity predicted to obtain a reliable field-based model (Faris et al., 2023b; 2024a).

## Constructing resilient models: investigating the development of 3D-QSAR

### Ligand preparation

Ensuring the accuracy and predicted activity of the 3D-QSAR and pharmacophore models depends on the precise alignment of molecules (Yadav et al., 2018). Before being converted into 3D structures, the molecular structures were originally in the 2D-SDF format.

The ligands were prepared using the LigPrep module of Schrödinger version 2021-3, which guaranteed the production of high-quality structures with the proper tautomeric forms, ring conformations, ionization states, and stereochemistry. To optimize their structures, all the molecules’ energies were minimized using the OPLS_2005 force field. The molecules were aligned using the Flexible Ligand Alignment Panel in Schrödinger version 2021-3 into Maestro, which offered the opportunity to carry out a flexible alignment for the chosen entries in the Project Table. The first chosen entry served as a template and was not altered. ConfGen was used to do a ligand torsional search on the following ligands (Ozgencil et al., 2020). Following a sequential alignment of the conformers produced by ConfGen with the reference ligand, the conformer that showed the best overlap with the reference ligand was chosen. It was necessary to replicate the original structures if you wanted to keep them, as this selected conformer superseded the previous entry. Well-minimized structures as input for flexible ligand alignment are advised. The template molecule with the highest pIC_50_ value must be taken into consideration. Implicit hydrogens are not permitted in the structures.

### Field-based approach

Over the years, researchers have come to emphasize quantitative structure-activity relationships (QSAR) for lead compound optimization. However, traditional QSAR methods typically only use imprecise estimates of three-dimensional structures. Maestro offers two approaches for QSAR modeling: field-based QSAR applies the ComFA/ComSIA approach, fitting and predicting properties using potential values on a grid, and atom-based QSAR uses atom types and their occupancy within a grid of cubes as independent variables. Make your choice in Maestro appropriately.

To create 3D-QSAR models, the PHASE module from Maestro—an interface to Schrödinger’s version 2021-3 utility—was used. Our goal was to create atom-based and field-based 3D-QSAR models to gain a better understanding of the relationship between structural features and biological activity. The models were created by randomly choosing a training set and a test set by the widely accepted 80:20 split suggested in the literature (Janet and Kulik, 2017; Jawarkar et al., 2023). To be sure the produced models were not the product of chance, we took additional measures and assessed them for statistical significance through both internal and external validation. Both active and inactive molecules were included in the training and test sets to guarantee the validity of the created models. We employed the same approach for MLR-based QSAR models, and we evaluated our models’ robustness in detail in each scenario. The dataset was split at random into 80% training and 20% test sets, and both 3D-QSAR models were trained using a PLS factor of 4. To guarantee that the molecules in the training and test sets were diverse, the software’s random selection process was visually confirmed. We stuck to a grid spacing of 1 Å for the chosen hypothesis. We created four field-based and four atom-based 3D-QSAR models.

### Assessing 3D-QSAR model predictive capability

We will explore the essential metrics utilized to assess 3D-QSAR models. These metrics offer crucial insights into the quality and reliability of models, guiding the compound optimization process. *R* ^2^ (coefficient of determination): This measures the proportion of the variance in the dependent variable explained by the independent variables, indicating an optimal fit when *R* ^2^ is close to 1. R^2^_CV_ (cross-validated coefficient of determination): Calculated through cross-validation methods, it assesses the model’s ability to generalize to independent data, similar to *R* ^2^. RMSE (root mean square error): This metric indicates the average of errors between predicted and observed values, providing an overall measure of model accuracy. Q^2^ (cross-validated coefficient of determination): Like R^2^_CV_, it evaluates how well the model predicts new, unseen data using cross-validation methods. These metrics, when applied, furnish a comprehensive understanding of the predictive power and reliability of 3D-QSAR models, thereby contributing to informed decision-making in compound design and optimization.

The evaluation of the 3D-QSAR model encompassed the scrutiny of key statistical parameters, which included the squared cross-validation coefficient (Q^2^), squared non-cross-validation coefficient (*R* ^2^), predictive *R* ^2^, and standard error of estimate (SEE). To gauge the internal quality of the developed model, particular attention was given to the Q^2^ value, with a criterion of >0.5 considered statistically significant (Faris et al., 2024c). The *R* ^2^ value served as a relative measure of the regression fit, and a value approaching 1.0 indicated a robust fit. Additionally, insights into the variation in residuals or the regression line were gleaned from the standard error of estimate (Clark et al., 1989; Shinde et al., 2017).

## Molecular docking (reversible)

Reversible (non-covalent) docking stands out as a prevalent approach in the realm of molecular docking, a computational technique utilized to forecast the binding affinity and orientation of a small molecule (ligand) within a receptor or target protein (Aljoundi et al., 2020; Faris et al., 2024b).

### Reversible (non-covalent)

Reversible (non-covalent) docking entails forecasting the non-covalent interactions between the ligand and the target protein. These interactions encompass hydrogen bonding, van der Waals forces, and hydrophobic, and electrostatic interactions. The primary goal of reversible docking is to predict the most favorable binding pose and affinity of the ligand within the target protein without forming a covalent bond. Widely employed in drug discovery and virtual screening, reversible docking aids in identifying potential lead compounds capable of binding to the target protein with high affinity and specificity. An advantage of reversible docking lies in the potential for ligand dissociation, facilitating the development of drugs with favorable pharmacokinetic properties.

Before conducting molecular docking, the ligands designated for docking underwent optimization using the Ligprep tool. Subsequently, we retrieved the structures of CDK9 and CYP3A4 from the RCSB database (PDB ***ID***: 7NWK and 8EWS). The crystal complex of 7NWK included water molecules and the co-crystallized ligand bound to the protein. For protein preparation, we removed all water molecules and co-crystallized ligands from 7NWK, and polar hydrogens were added to the CDK9 protein structure using Discovery Studio software 2021. Similar steps were applied to 8EWS, with the exception that the included ligands were retained, as they are integral to the metabolism of CYP3A4. Following the preparation of ligands and proteins, molecular docking was executed using Autodock4 and Autodock-Vina to explore the active site of 7NWK and 8EWS, determined by the region encompassing the co-crystallized ligands (Morris et al., 2008). The three-dimensional grid was established using the AUTOGRID algorithm, which calculates the binding energy between ligands and their receptor. The default grid size for CDK9 (7NWK) and CYP3A4 (8EWS) with new compounds was configured as x = 60, y = 60, and z = 60, with a spacing of 0.375 Å between grid points. The center of the grid corresponds to the active site of the receptors CDK9-4LH and CYP3A4, specified by coordinates (x = −42.62 Å, y = −39.29 Å, and z = −2.86 Å) and (x = −-15.37 Å, y = −30.74 Å, and z = −10.29 Å), respectively.

## ADME-TOX

ADMET analysis, medicinal chemistry, and the evaluation of lead-like and drug-like properties were performed using easily accessible online tools. One such tool employed for these analyses is SwissADME (Daina et al., 2017) and pkCSM (Pires et al., 2015); assessing drug candidates and compounds involves evaluating their potential toxicity for human use.

## Molecular dynamics simulation

The newly created compounds, which exhibited enhanced binding affinity with CDK9 and CYP3A4, underwent all-atom molecular dynamics simulations using GROMACS 2021 (Groningen Machine for Chemical Simulation) software (Van Der Spoel et al., 2005; Abraham et al., 2015). Before initiating the MD simulations, the CHARMM-GUI web server (Jo et al., 2008) was employed to generate the initial input parameters, implementing the CHARMM36 force field (Huang and MacKerell Jr, 2013; Ziada et al., 2022; Faris et al., 2023a, n. d.). The simulation was conducted at a pH of 7. Before entering the production phase, each complex was solved within a rectangular grid box, surrounded by TIP3P water molecules, and supplemented with the requisite counter-ions (Na^+^, Cl^−^) to maintain a salt concentration of 0.15 M, achieved through Monte Carlo ion displacement. Energy minimization was executed for each system using the steepest descent algorithm, encompassing a maximum of 50,000 steps and a maximum force of 10.0 kJ/mol. The temperature and atmospheric pressure were set to 310 K and 1.01325 bar, respectively. For NVT equilibration, two stages were carried out, each lasting 10 ns. Canonical (NVT) and isothermal-isobaric (NPT) ensembles were utilized to equilibrate each system. Subsequently, MD simulations were conducted for a duration of 100 nanoseconds. To assess the structural stability of the designed molecules, various parameters, including root mean square deviation (RMSD), the radius of gyration (RoG), solvent accessible surface area (SASA), and root mean square flexibility (RMSF), were analyzed based on the dynamics trajectory results.

## Molecular Mechanics/Poisson-Boltzmann surface area (MM/PBSA)

The MM/PBSA calculation is a powerful method for estimating the binding energy between a ligand molecule and a protein. This method combines molecular mechanics (MM) calculations and Poisson-Boltzmann (PB) computations to account for both stoichiometric and electrostatic interactions between molecular components. The outcomes of these calculations can assist in understanding the underlying forces of molecular bonding, which is essential for drug design, exploring new therapies, and studying protein structure and dynamics. In this study, the calculation was performed using the latest version of the gmx_MMPBSA tool (Valdés-Tresanco et al., 2021).

Various terms of energy calculated by MM/PBSA include (Miller et al., 2012; Valdés-Tresanco et al., 2021): Energy (kcal/mol): The total energy calculated by MM/PBSA, which is the sum of all other terms listed below. ΔV_DWAALS_: The variation in van der Waals energy, reflecting weak interactions between atoms due to induced dipole charges. ΔE_EL_: The variation in long-range electrostatic energy, representing electrostatic interactions between fixed atomic charges. Δ_EPB_: The variation in polarizable solvent energy, considering the solvent effect on charges and dipole moments. ΔG_SOLV_: The variation in solvent energy, measuring the solvent effect on the overall energy of the molecule. Δ_TOTAL_: The total variation in energy, which is the sum of all preceding terms.

These terms offer a detailed view of individual contributions to binding energy, thus allowing for a fine analysis of the forces that maintain the ligand molecule’s bound conformation to the protein. Interpretation of these terms can aid in identifying critical binding sites, understanding bonding mechanisms, and guiding the development of new ligands with desirable properties.

## Data Availability

Data are contained within the Supplementary Material.
