# Supplementary material for: Development of novel CDK9 and CYP3A4 inhibitors for cancer therapy through field and computational approaches
Source: Front Chem. 2024 Oct 21;12:1473398. doi: 10.3389/fchem.2024.1473398 (PMC11532072; doi:10.3389/fchem.2024.1473398)
Supplement: Supplementary file 1 [file DataSheet1.docx]

Development of novel CDK9 and CYP3A4 inhibitors for cancer therapy through field and computational approaches

Aisha A. Alsfouk* ^1^, Abdelmoujoud Faris ^2^*, Ivana Cacciatore ^3^, Radwan ِAlnajjar ^4, 5, 6^

1. Department of Pharmaceutical Sciences, College of Pharmacy, Princess Nourah bint Abdulrahman University, Riyadh, 11671, Saudi Arabia. [aaalsfouk@pnu.edu.sa](mailto:aaalsfouk@pnu.edu.sa)
2. LIMAS, Department of Chemical Sciences, Faculty of Sciences Dhar El Mahraz, Sidi Mohamed Ben Abdellah University, Fez 30000, Morocco. [abdelmoujoud.faris@usmba.ac.ma](mailto:abdelmoujoud.faris@usmba.ac.ma)
3. Department of Pharmacy, University ‘G. d’Annunzio’ of Chieti-Pescara, Italy. [ivana.cacciatore@unich.it](mailto:ivana.cacciatore@unich.it)
4. CADD Unit, PharmD, Faculty of Pharmacy, Libyan International Medical University, Benghazi, Libya. Radwan.alnajjar@uob.edu.ly

Corresponding author *: [aaalsfouk@pnu.edu.sa](mailto:aaalsfouk@pnu.edu.sa), [abdelmoujoud.faris@usmba.ac.ma](mailto:abdelmoujoud.faris@usmba.ac.ma)


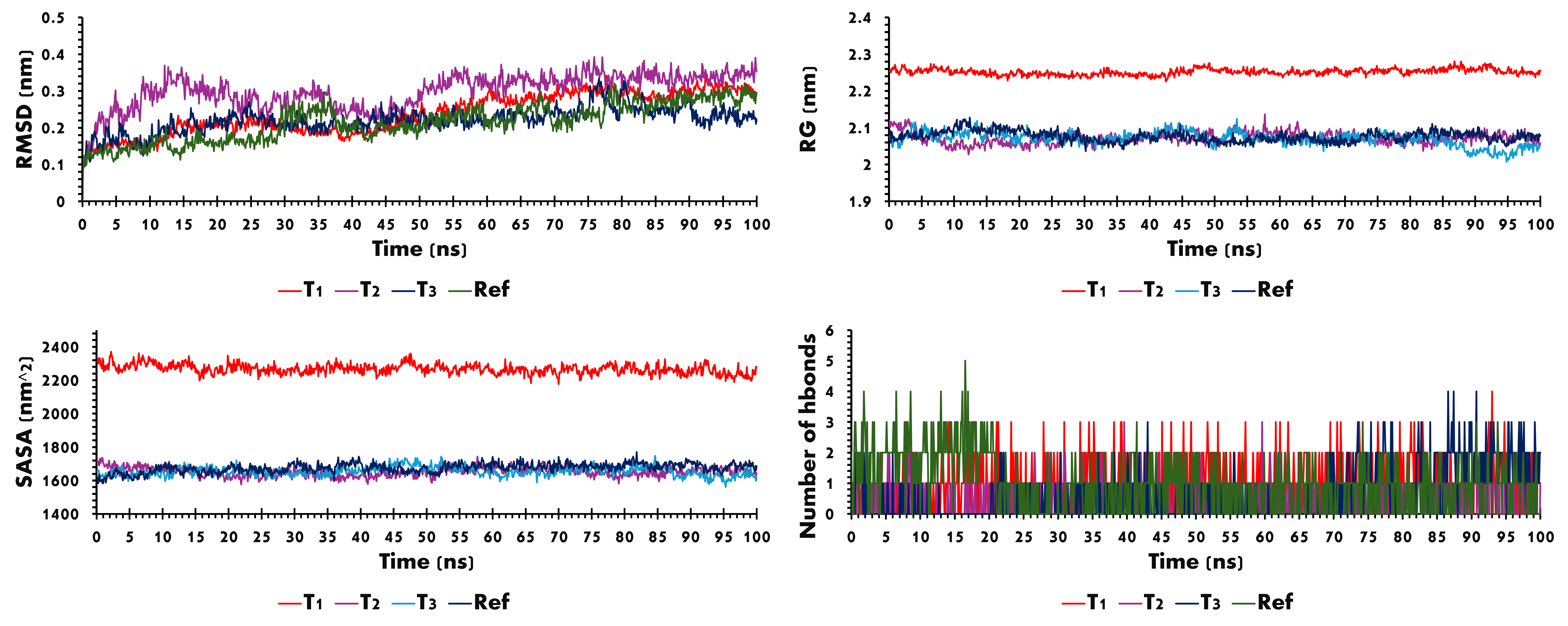


**Figure S1.** Plots depicting the analyses of RMSD, Rg, SASA, and Hbonds for new compounds T1, T2, T3, and Reference.

.
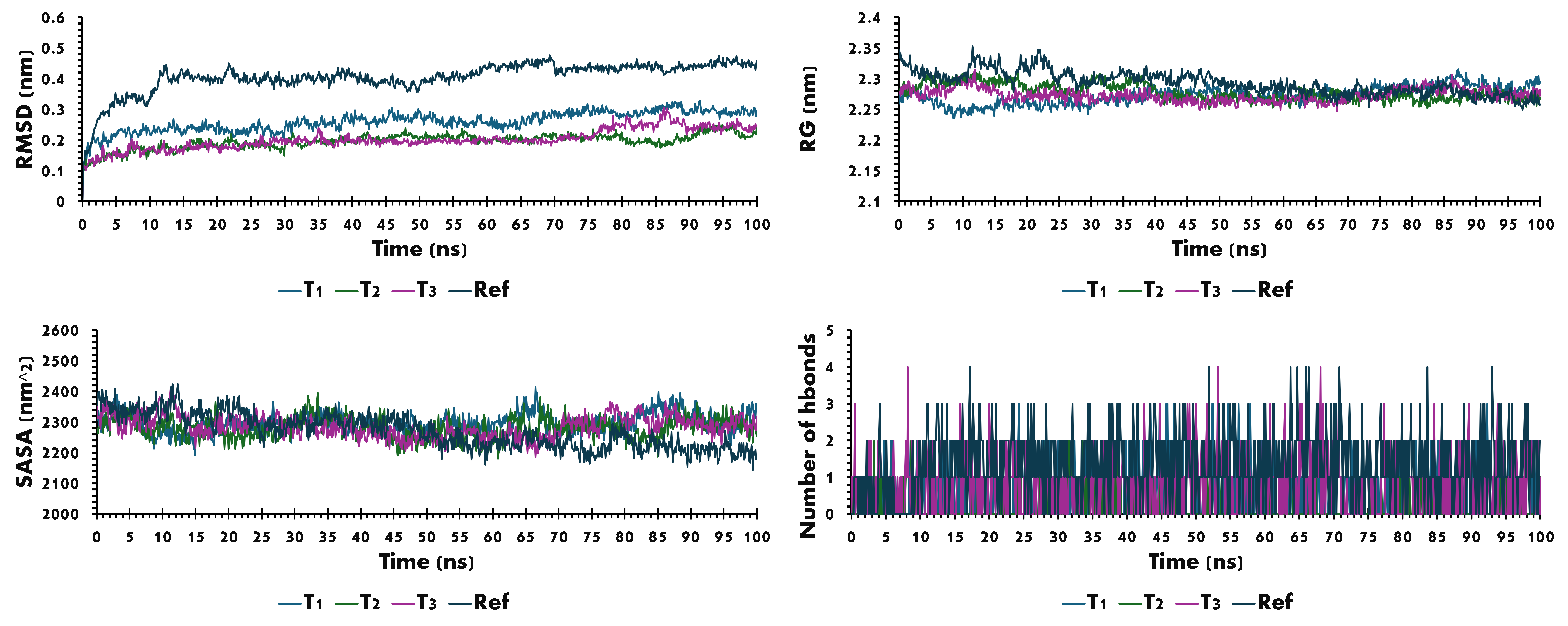


**Figure S2.** Plots depicting the analyses of RMSD, Rg, SASA, and Hbonds for new compounds T1, T2, T3, and Reference.


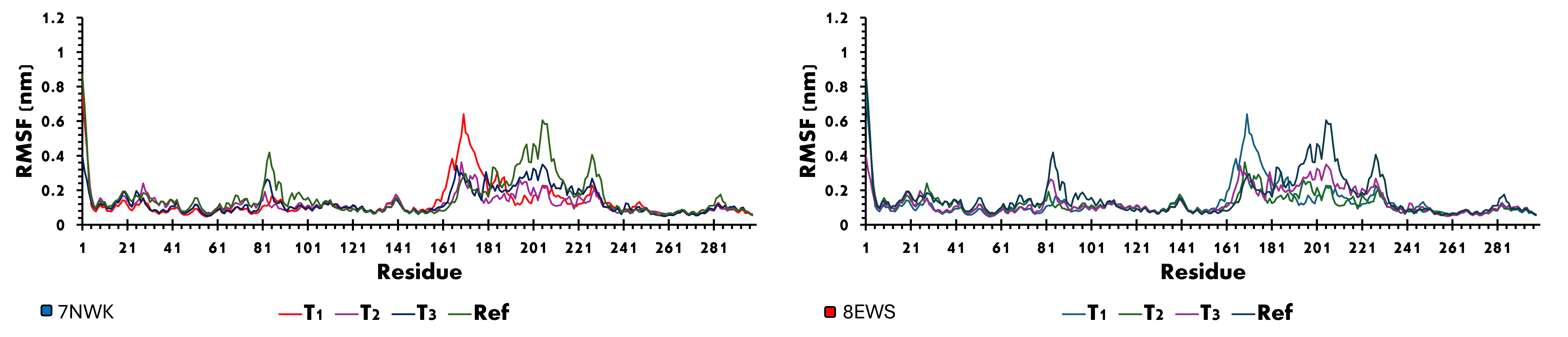


**Figure S3.** Plots depicting the analyses of RMSF new compounds with CDK9 and CYP3A4.


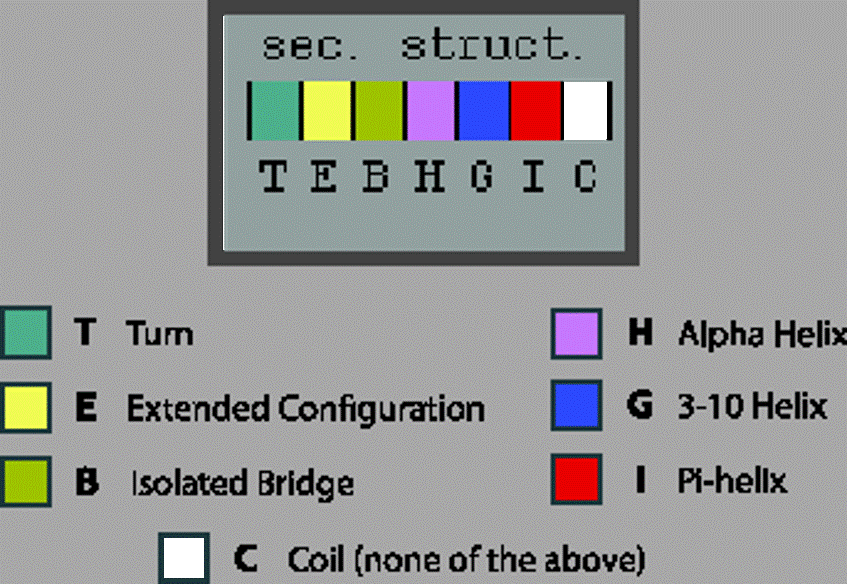


**Figure S4.** Secondary structure maps with color key.


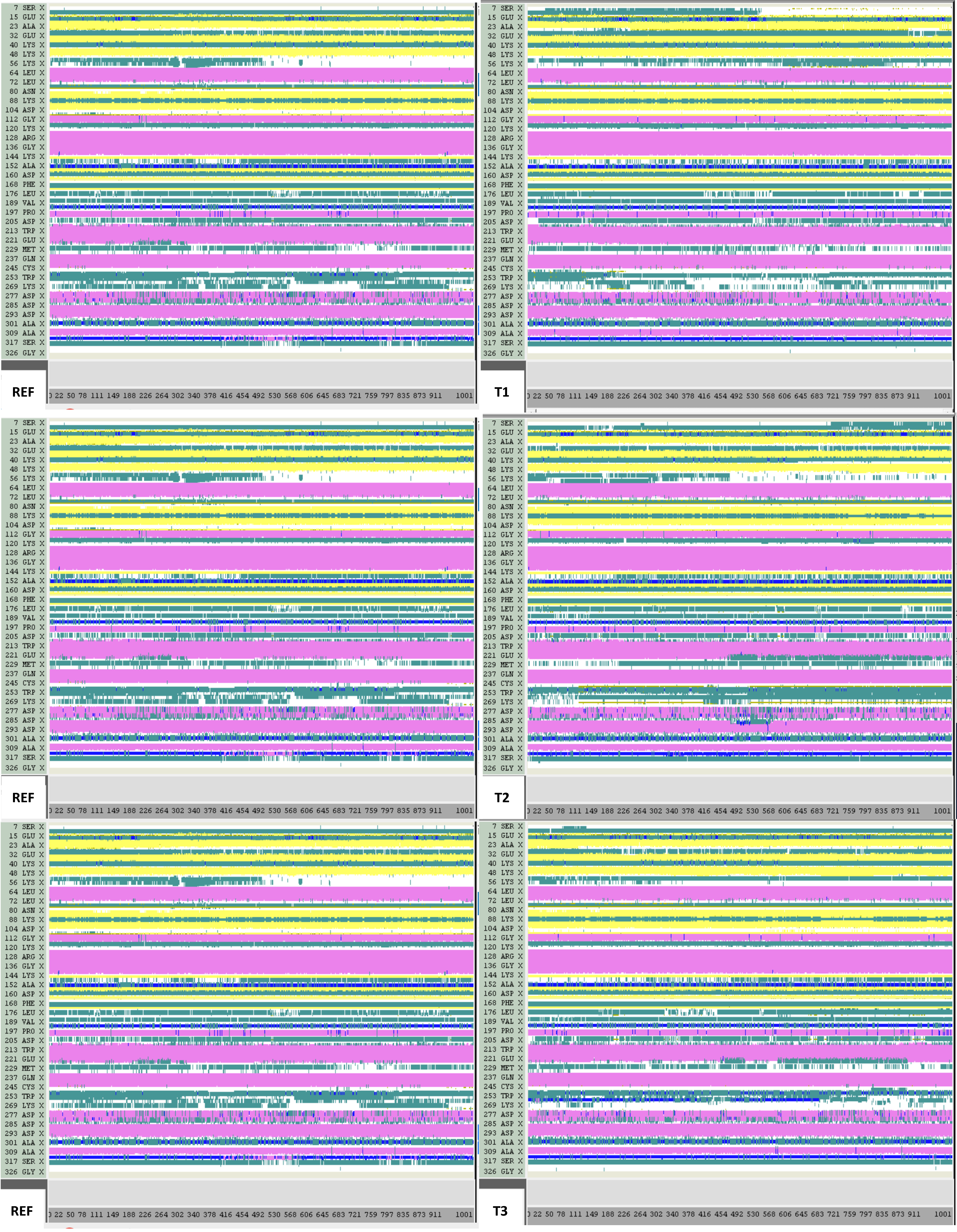


**Figure S5.** DSSP comparison between new compound and reference.


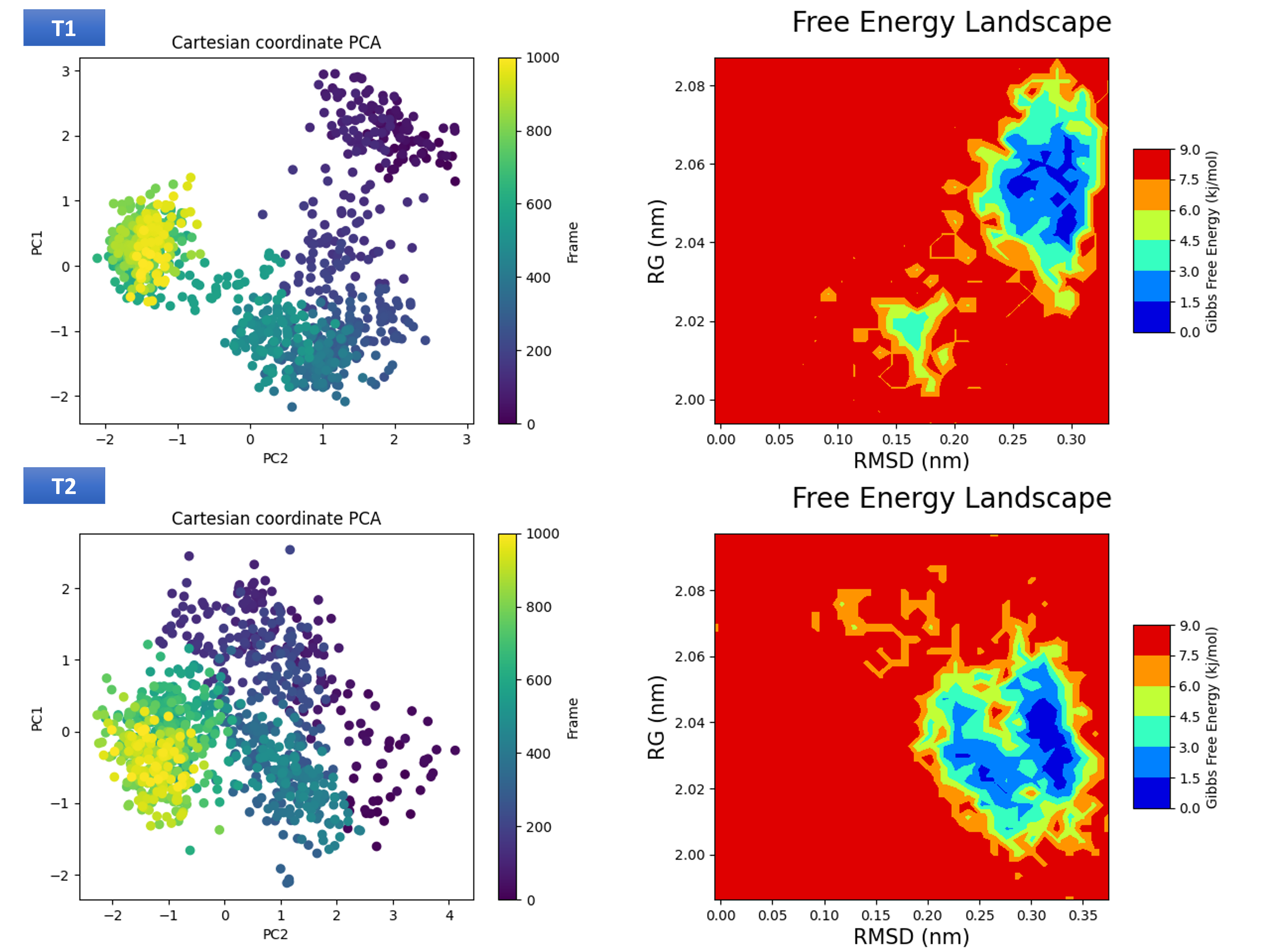


**Figure S6.** Analyses of New Compounds T1 and T2 Using PCA and FEL.


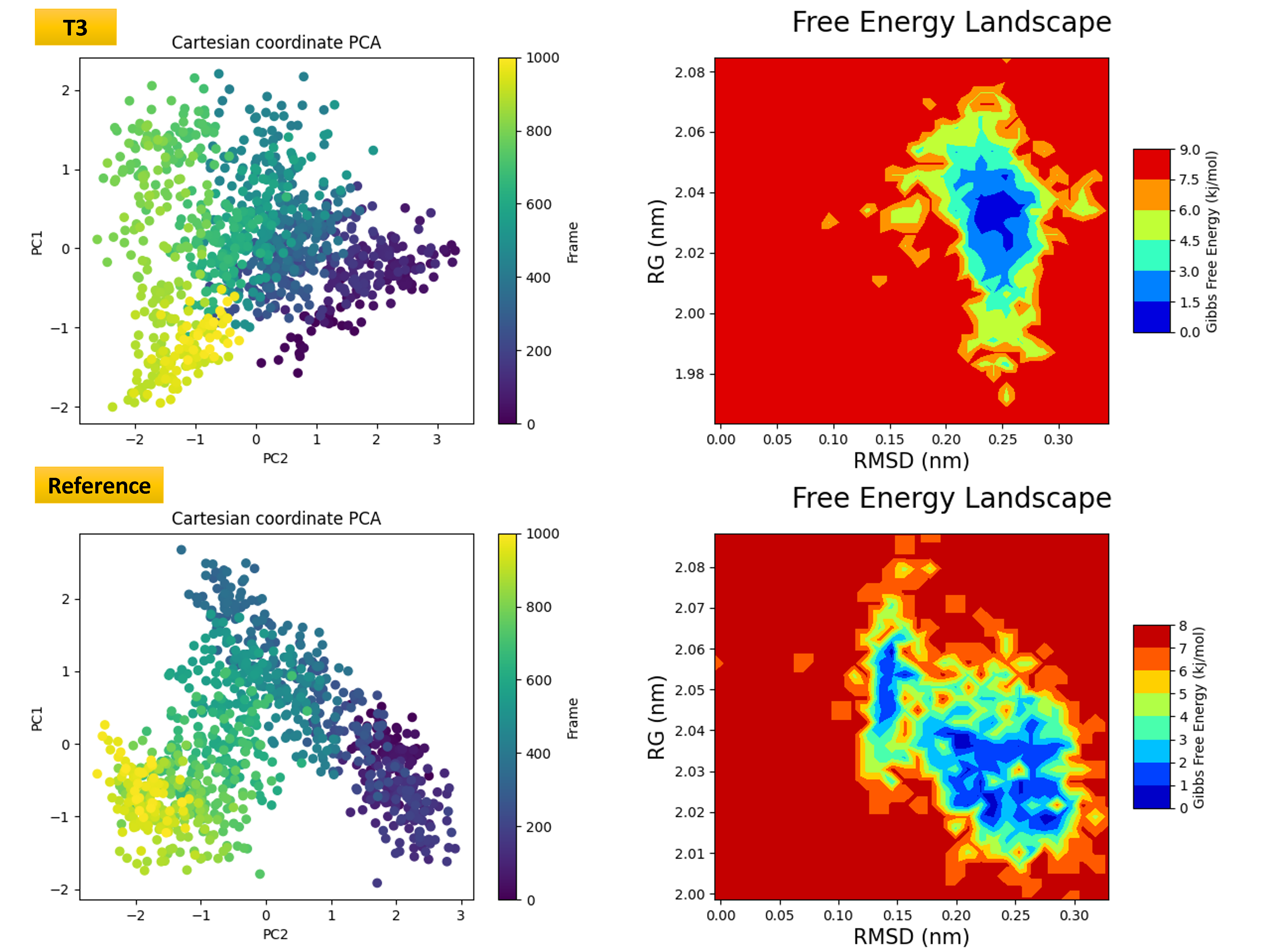


**Figure S7.** Analyses of New Compounds T1, and reference Using PCA and FEL.


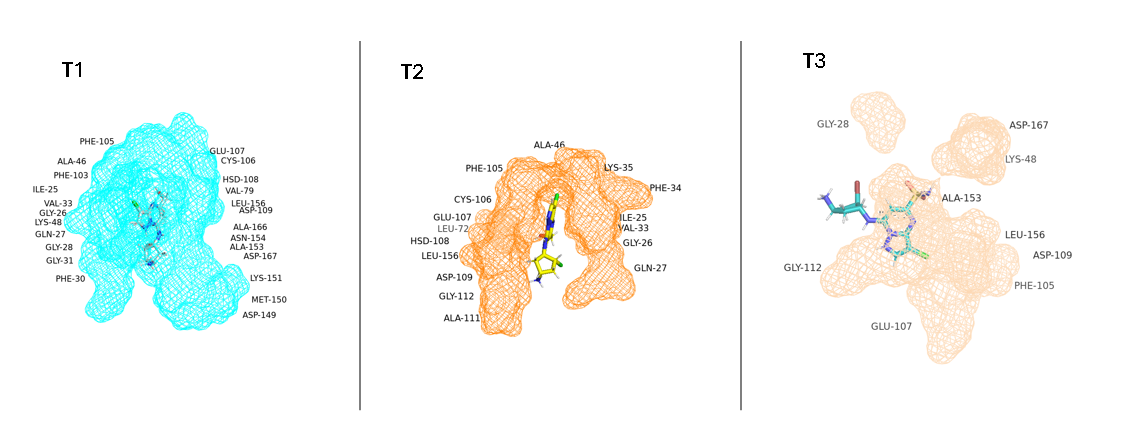


**Figure S8.** The most stable conformations of the new compound with CDK9 correspond to the energy minima for the new inhibitors.


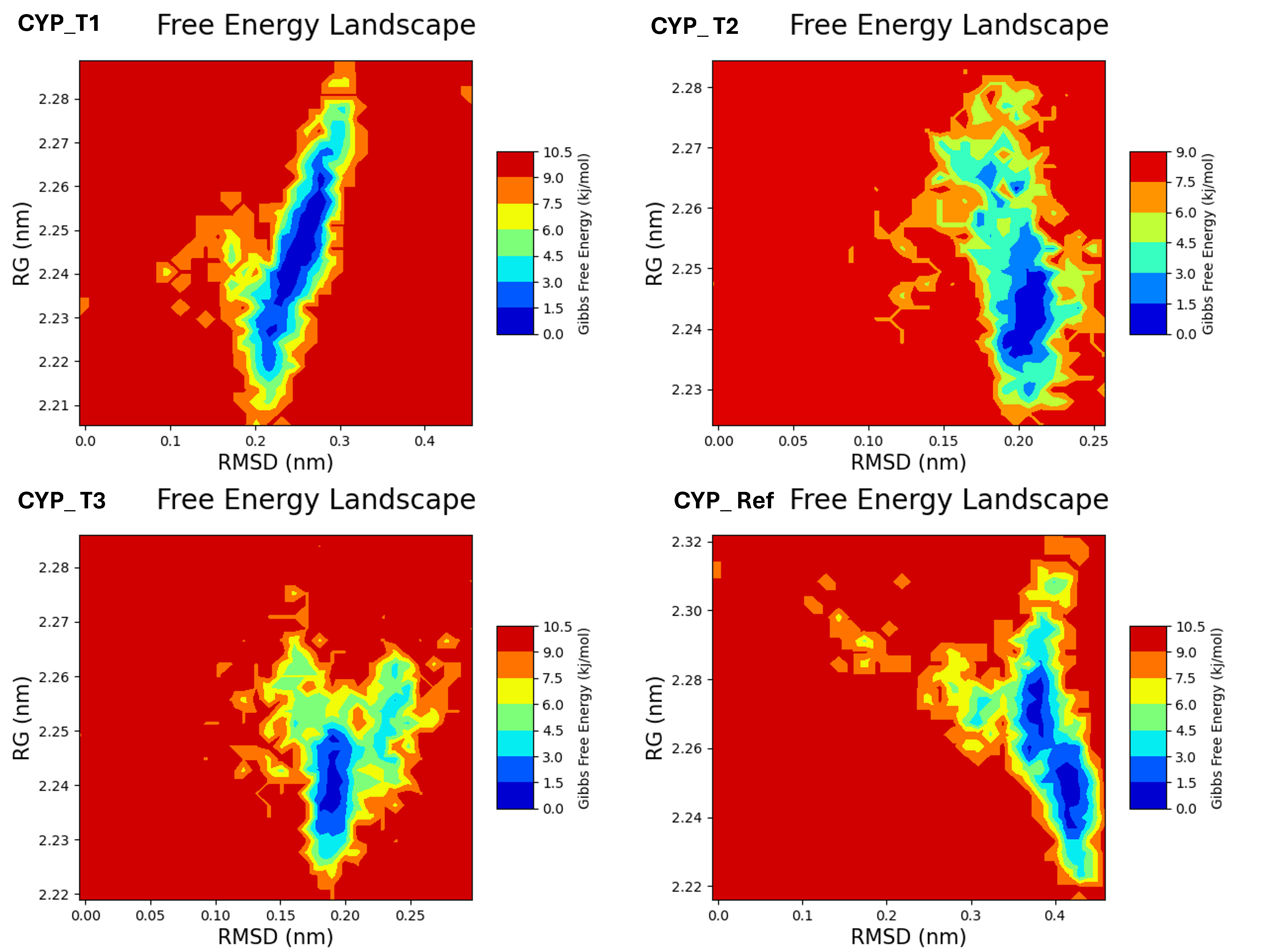


**Figure S9.** Analyses of New Compounds T1-3 and Ref with CYP3A4 Using PCA and FEL
